# Calcium-Dependent Dephosphorylation of the Histone Chaperone DAXX Regulates H3.3 Loading and Transcription upon Neuronal Activation

**DOI:** 10.1016/j.neuron.2012.02.021

**Published:** 2012-04-12

**Authors:** David Michod, Stefano Bartesaghi, Amel Khelifi, Cristian Bellodi, Laura Berliocchi, Pierluigi Nicotera, Paolo Salomoni

**Affiliations:** 1 Samantha Dickson Brain Cancer Unit, University College London Cancer Institute, London WC1E 6DD, UK; 2 Medical Research Council Toxicology Unit, Leicester LE1 9HN, UK; 3 Helen Diller Comprehensive Cancer Center, UCSF, San Francisco, CA 94143-0875, USA; 4 German Center for Neurodegenerative Diseases (DZNE), Bonn 53175, Germany

## Abstract

Activity-dependent modifications of chromatin are believed to contribute to dramatic changes in neuronal circuitry. The mechanisms underlying these modifications are not fully understood. The histone variant H3.3 is incorporated in a replication-independent manner into different regions of the genome, including gene regulatory elements. It is presently unknown whether H3.3 deposition is involved in neuronal activity-dependent events. Here, we analyze the role of the histone chaperone DAXX in the regulation of H3.3 incorporation at activity-dependent gene loci. DAXX is found to be associated with regulatory regions of selected activity-regulated genes, where it promotes H3.3 loading upon membrane depolarization. DAXX loss not only affects H3.3 deposition but also impairs transcriptional induction of these genes. Calcineurin-mediated dephosphorylation of DAXX is a key molecular switch controlling its function upon neuronal activation. Overall, these findings implicate the H3.3 chaperone DAXX in the regulation of activity-dependent events, thus revealing a new mechanism underlying epigenetic modifications in neurons.

## Highlights

► Neuronal activation induces H3.3 loading at regulatory elements of IEGs ► H3.3 deposition at regulatory regions of selected IEGs is dependent on DAXX ► DAXX regulates transcriptional induction of selected IEGs ► DAXX function is under the control of calcineurin

## Teaser

Epigenetic modification can occur through deposition of histone variants. Michod et al. propose a mechanism underlying dynamic, activity-dependent replacement of the histone variant H3.3 and transcriptional regulation in the central nervous system.

## Introduction

Activity-dependent modifications of chromatin in neurons are believed to contribute to dramatic changes in neuronal circuitry (Riccio, 2010). Calcium entry into the postsynaptic neuron leads to transcriptional activation through induction of signaling cascade involving key kinases and phosphatases, such as Ca^2+^/calmodulin-dependent kinases and calcineurin. A number of activity-responsive genes, such as the neurotrophin *Bdnf*, are kept in a repressive state through mechanisms involving the recruitment of coREST, histone deacetylases HDAC1/2, and the methyl-CpG-binding protein 2 (MeCP2) (Ballas et al., 2005; Chen et al., 2003a; Martinowich et al., 2003). After synaptic stimulation, HDAC2 (and possibly HDAC1) is nitrosylated, leading to its inactivation (Nott et al., 2008), whereas calcium-dependent phosphorylation of MeCP2 causes the dissociation of the corepressor complex from the *Bdnf* promoter (Chen et al., 2003a; Martinowich et al., 2003; Zhou et al., 2006).

Several regulators of activity-dependent transcription have been implicated in human disorders of the central nervous system (CNS). For instance, mutations of the *MeCP2* gene cause Rett syndrome (Amir et al., 1999). MeCP2 is found in a complex containing the proteins ATRX and cohesin, which are mutated in the ATR-X and CdLS syndromes, respectively (Gibbons et al., 1995; Kernohan et al., 2010; Liu and Krantz, 2008; Nan et al., 2007). Although clearly distinct from one another, many of these disorders share similar clinical features, thus suggesting that common symptoms may be caused by underlying interlinked molecular mechanisms. ATRX interacts with the chromatin-associated protein DAXX, which was originally cloned as a FAS-associated protein (Yang et al., 1997). However, subsequent studies have revealed that in primary cells, DAXX is mainly nuclear (Lindsay et al., 2009). Both DAXX and ATRX are found to be associated with heterochromatic foci and promyelocytic leukemia nuclear bodies (PML-NBs; Ishov et al., 2004; Lallemand-Breitenbach and de Thé, 2010; Salomoni and Betts-Henderson, 2011; Xue et al., 2003; Zhu et al., 2005). PML is a tumor suppressor involved in the t(15;17) translocation of acute promyelocytic leukemia. We have recently shown that PML controls cell fate in neural progenitors during cortical development (Regad et al., 2009). DAXX interacts with transcription factors and chromatin modifiers, which include histone deacetylases, the histone acetyl-transferase CBP, and DNA methyltransferases (Hollenbach et al., 2002; Kuo et al., 2005; Puto and Reed, 2008; Salomoni and Khelifi, 2006).

Recent studies have proposed a more direct role for DAXX in chromatin remodeling through regulation of histone loading. In particular, DAXX has been shown to act as a histone chaperone for the histone variant H3.3 (Drané et al., 2010; Lewis et al., 2010). Unlike H3.1 and H3.2, H3.3 is loaded onto DNA in a replication-independent manner. These histone variants are conserved to lower eukaryotes and are believed to be important carriers of epigenetic information (Hake and Allis, 2006; Szenker et al., 2011). DAXX and ATRX interact with H3.3 and mediate H3.3 loading onto telomeres and pericentric heterochromatin (Drané et al., 2010; Goldberg et al., 2010; Lewis et al., 2010). DAXX is required for H3.3/ATRX binding (Drané et al., 2010). Recent studies showed that H3.3, DAXX, and ATRX are found mutated in the brain tumor glioma (Schwartzentruber et al., 2012; Wu et al., 2012), thus suggesting that alterations of H3.3 loading could contribute to cancer pathogenesis in the central nervous system. Loading of H3.3 at transcription start site (TSS) and gene bodies of transcriptionally active loci is dependent on the chaperone HIRA (Goldberg et al., 2010). Notably, H3.3 is also enriched at regulatory regions not immediately adjacent to TSS (Goldberg et al., 2010; Jin et al., 2009; Mito et al., 2007). Deposition at those sites has been proved in part to be HIRA and ATRX independent (Goldberg et al., 2010). It has been speculated that DAXX may mediate H3.3 loading at regulatory regions through its association with the histone chaperone DEK (Elsaesser and Allis, 2010; Sawatsubashi et al., 2010), but evidence for this function is still lacking. Although chromatin relaxation at actively transcribed genes has been proposed to promote H3.3 loading (Henikoff, 2008), it is presently unknown whether neuronal activity-dependent transcription influences deposition of this histone variant. We set out to study H3.3 deposition at activity responsive genes and to determine whether DAXX represents one of the chaperones responsible for this activity.

Here, we show that upon neuronal activation, DAXX mediates H3.3 loading at regulatory regions of selected immediate early genes and contributes to their transcriptional induction. The histone chaperone activity of DAXX is controlled by a calcium- and calcineurin-dependent phosphorylation switch. This work implicates DAXX as one of the chaperones for H3.3 deposition at regulatory regions in neurons. In addition, it proposes a mechanism regulating chromatin variations upon neuronal activation.

## Results

### DAXX Is Expressed in the Mouse CNS

**Figure 1:**
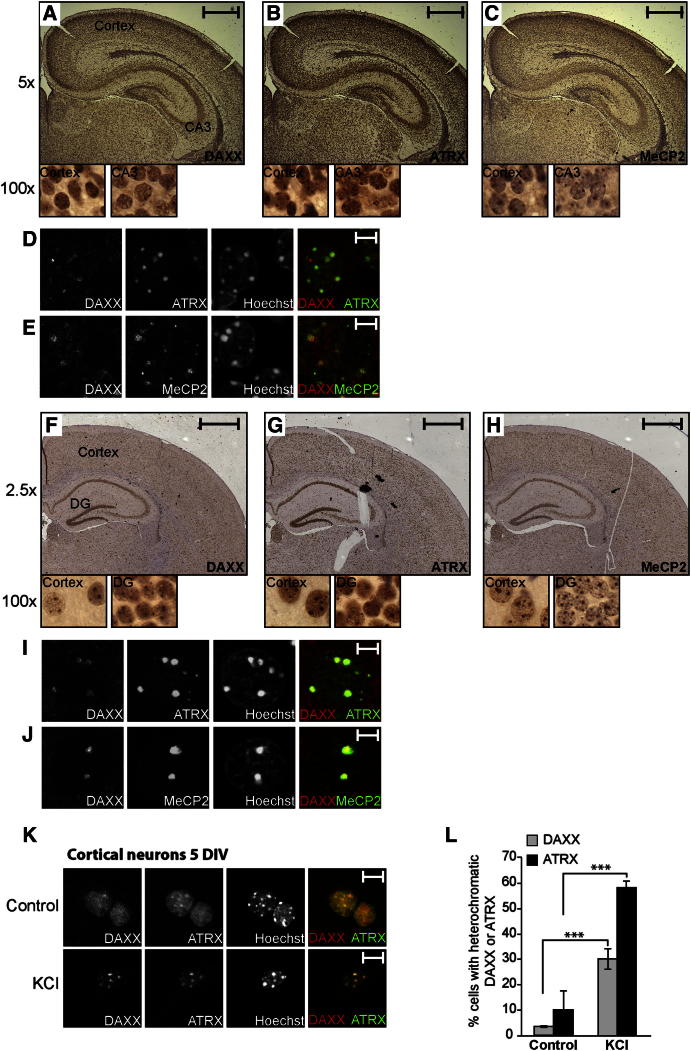
DAXX Is Expressed in the Mouse Postnatal Brain and Localizes to the Nucleus (A–C) Immunohistochemistry of DAXX, ATRX, and MeCP2 on coronal sections of the mouse brain on P2. Higher-magnification images of a cortex area and CA3 (cornu ammonis) region of the hippocampus are shown in insets. Scale bars represent 500 μm. (D and E) DAXX/ATRX and DAXX/MeCP2 immunofluorescence staining of a cortex area from coronal sections of the mouse brain at P2. Scale bars represent 5 μm. (F–H) Immunohistochemistry on coronal sections of the mouse brain at P30. Higher-magnification images of a cortex area and dentate gyrus (DG) region of the hippocampus are shown in insets. Scale bars represent 1,000 μm. (I and J) DAXX/ATRX and DAXX/MeCP2 immunofluorescence of a cortex area on coronal section of the mouse brain at P30. Scale bars represent 5 μm. (K) Immunofluorescence analysis of DAXX and ATRX localization in 5 DIV cortical neurons that were left untreated or were treated with 50 mM KCl for 3 hr. Scale bars represent 20 μm. (L) Quantification of DAXX and ATRX relocalization to heterochromatin. Data are mean ± SEM; n = 3; ^∗∗∗^p < 0.001; two-way analysis of variance (ANOVA) test with Bonferroni posttest.

We first analyzed the expression of DAXX in the embryonic and postnatal mouse brain. DAXX protein was detected as early as embryonic day 12.5 (E12.5) in the neuroepithelium (ventricular zone, VZ; see Figure S1A available online). At E17.5, DAXX expression became more pronounced in postmitotic cells of the cortical plate (CP) (Figure S1E). Early postnatally (postnatal day 2 [P2]) and in the adult brain (P30), DAXX was expressed both in the cortex and in the hippocampus (Figures 1A and 1F). At all stages, DAXX localized to the nucleus, where it was in part associated with heterochromatic foci and colocalized with ATRX (VZ and CP) and the ATRX-interacting protein MeCP2 (CP) (Figures 1A–1J and S1A–S1H) (Nan et al., 2007). DAXX and ATRX interacted in whole-brain extracts (Figure S1I), whereas we failed to detect interaction between DAXX and MeCP2 (data not shown). In primary cultures of cortical neurons, DAXX was nuclear and displayed colocalization with ATRX and MeCP2, especially starting from 5 days in vitro (5 DIV; Figures S1J and S1K; data not shown). The promyelocytic leukemia protein was absent from 5 DIV cultures (data not shown). We next tested whether membrane depolarization, which mimics neuronal activation, affects DAXX subnuclear distribution. To this end, we exposed 5 DIV cortical neurons to high potassium chloride (50 mM KCl) and analyzed DAXX localization. As shown in Figures 1K and 1L, the degree of DAXX and ATRX colocalization increased shortly following depolarization. These changes in localization were not associated with increased expression of the two proteins (Figure S1L; see also Figure 5B). As reported previously (Martinowich et al., 2003), MeCP2 followed the same pattern of relocalization (data not shown). Taken together, these data show that DAXX displays a nuclear distribution in neurons and colocalizes with both ATRX and MeCP2.

### DAXX Associates with Regulatory Elements of Selected Activity-Regulated Genes

**Figure 2:**
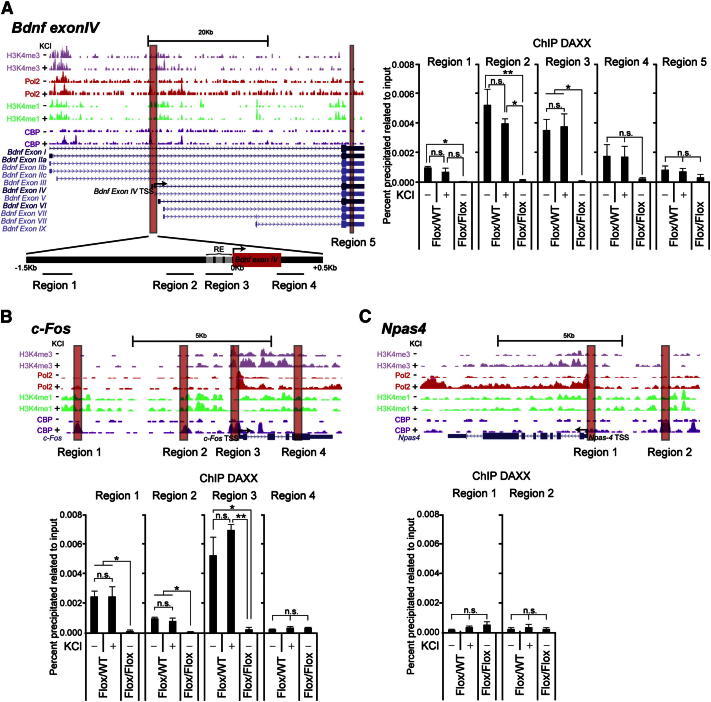
DAXX Is Present at Regulatory Elements of Selected Activity-Regulated Genes (A) Representative Genome Browser image of regulatory element marks for the *Bdnf* gene in unstimulated and membrane-depolarized neurons (2 hr). H3K4me3 marks promoter regions, whereas H3K4me1 marks enhancer regions. CBP and PolII have been shown to mark both these regions upon neuronal activation. The gene map and the location of primers are shown underneath (regions 1–5). ChIP analysis of DAXX enrichment at the selected regions in *DAXX^Flox/WT^* cortical neurons in the absence or presence of 50 mM KCl (3 hr). We performed ChIP by using CRE-infected *DAXX^Flox/Flox^* cells as background control. (B and C) Same as in (A) for *c-Fos* and *Npas4*. Data are mean ± SEM; n = 3 or n = 6 for *Bdnf Exon IV* region 4; n.s., not significant; ^∗^p < 0.05; ^∗∗^p < 0.01; two-way ANOVA test with Bonferroni posttest.

We next investigated whether DAXX could associate with chromatin in neurons. Neuronal activation triggers rapid chromatin changes at a number of immediate early genes (IEGs) (Greer and Greenberg, 2008; Saha et al., 2011). We started by studying the *Bdnf* gene. Of the eight *Bdnf* promoter regions, the promoter IV is highly responsive to neuronal activity in cultured cortical neurons (Tao et al., 1998). The key regulatory elements responsible for the calcium-dependent expression of *Bdnf Exon IV* have been previously characterized (RE, calcium-responsive element in Figure 2A) (Chen et al., 2003b; Tao et al., 1998, 2002). Furthermore, a recent report has annotated the main neuronal activity-regulated enhancer and promoter regions in neurons by using chromatin immunoprecipitation followed by sequencing (ChIP-seq) (Kim et al., 2010). We selected the promoter regions of *Bndf Exon IV* that encompass the calcium regulatory elements (regions 2 and 3) and two more distal regions (regions 1 and 4; Figure 2A). We failed to identify distal enhancer regions positive for the enhancer mark histone 3 monomethylated lysine 4 (H3K4me1), probably due to the complex organization of the *Bdnf* gene. We used ChIP to investigate the association of endogenous DAXX with the different proximal and more distal regions of *Bdnf Exon IV*. As a negative control, we used cortical neurons derived from a conditional *DAXX* knockout mouse model (*DAXX^Flox/Flox^*; Figures S2A and S2B), in which expression of the CRE recombinase abrogates DAXX expression (Figures S2C–S2F). Among the regions examined, DAXX-associated chromatin was enriched in sequences proximal to the TSS (regions 2 and 3) (Figure 2A). Although binding to region 4 was also detected, it did not reach statistical significance over CRE-infected *DAXX^Flox/Flox^* cells (Figure 2A). Moreover, we failed to detect significant association to the transcribed region (region 5; Figure 2A). No binding was detected when we used chromatin from CRE-infected *DAXX^Flox/Flox^* cells (Figure 2A). We concluded that, in cultured neurons, DAXX is predominantly associated with sequences at or adjacent to the TSS of *Bdnf Exon IV*. We then investigated MeCP2 association with the *Bdnf Exon IV* regulatory regions. MeCP2 was found at proximal promoter regions (2 and 3) in the absence of KCl (Figure S2G), whereas association with regions 1 and 4 was negligible (Figure S2G). Thus, DAXX and MeCP2 are enriched at overlapping *Bdnf Exon IV* regulatory regions. Neuronal activation caused the release of MeCP2 from the promoter (Figure S2G), as previously reported (Chen et al., 2003a; Martinowich et al., 2003), but it did not affect DAXX association (Figure 2A).

We then examined whether DAXX is present at regulatory elements of two additional IEGs, *c-Fos* and *Npas4* (Greenberg et al., 1986; Lin et al., 2008). Based on the abovementioned ChIP-seq study (Kim et al., 2010), we selected two enhancer regions (regions 1 and 2, corresponding to e4 and e3 in Kim et al., 2010), the promoter (region 3) and transcribed (region 4) regions of *c-Fos* (Figure 2B). DAXX was found highly enriched at sequences encompassing the promoter region (Figure 2B; region 3). DAXX-deleted cells were used as negative control (see above). A significant association with both enhancer regions was also detected (Figure 2B; regions 1 and 2). However, we failed to reveal any significant interaction with the transcribed region of *c-Fos* (Figure 2B; region 4). With respect to the *Npas4* gene, we next analyzed DAXX association with two regulatory regions (regions 1 and 2; Figure 2C), which have features of promoter and enhancer, respectively. We failed to detect DAXX association with any of the *Npas4* regulatory elements analyzed (Figure 2C). This apparent selectivity of binding prompted us to extend our analysis to additional IEGs (*Zif 268*, *Nurr1*, *Ier2*, *Gadd45g*, *Egr2*, *Dusp6*, and *Arc*), which had been previously described to respond to neuronal activation (Saha et al., 2011). Two of them, the transcription factor *Egr2* and the serine/threonine/tyrosine phosphatase *Dusp6*, were enriched in DAXX immunoprecipitates (Figure S2H). Overall, DAXX association with *c-Fos*, *Egr2*, and *Dusp6* was not affected by KCl treatment (Figures 2B and S2H).

We next investigated whether the DAXX-interacting protein ATRX displays similar selectivity for IEG regulatory regions. Indeed, ChIP analysis showed that ATRX interacts with the *Bdnf* and *c-Fos* regulatory elements, but it failed to bind the *Npas4* gene (Figure S2I). We confirmed that DAXX and ATRX could interact in isolated cortical neurons (Figure S2J). KCl treatment did not affect this interaction or ATRX association with *Bdnf* and *c-Fos* regulatory regions (Figures S2I and S2J). Thus, DAXX and ATRX interact in neurons and display similar binding selectivity for IEG regulatory elements.

### Activation-Induced H3.3 Loading at Selected Immediate Early Genes Is Dependent on DAXX

**Figure 3:**
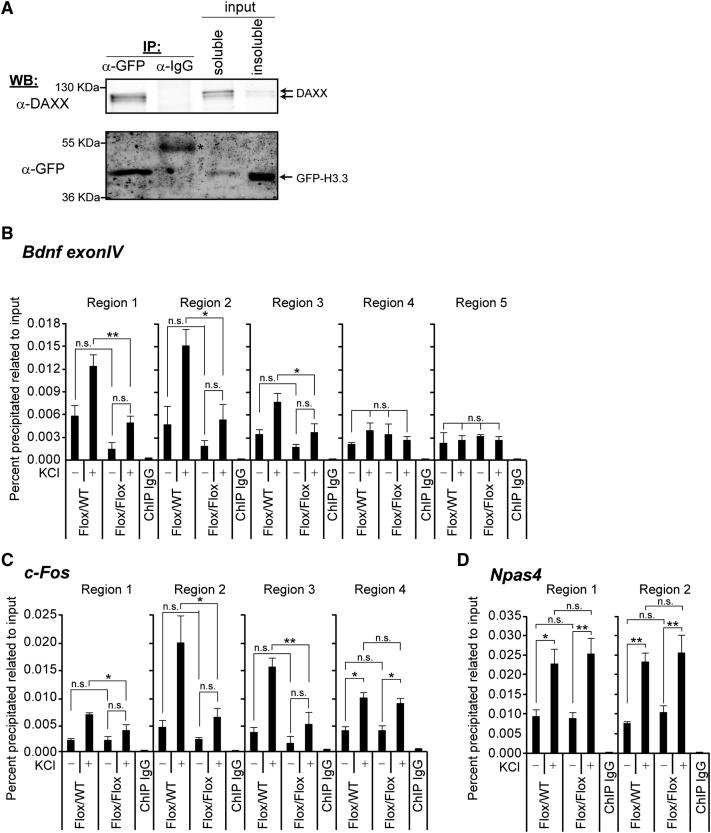
DAXX Regulates Deposition of H3.3 at Regulatory Regions of *Bdnf Exon IV* and *c-Fos* (A) Interaction between DAXX and H3.3 in cortical neurons. Extracts from 4 DIV cortical neurons, infected with H3.3-YFP lentivirus, were immunoprecipitated (IP) with anti-GFP or a control antibody. The immunoprecipitates were analyzed by western blotting using the indicated antibodies. Arrows indicate different DAXX migration forms. Asterisk indicates IgG heavy chain. (B–D) ChIP analysis of H3.3 enrichment at the regions analyzed in Figure 2 was performed by using chromatin from *DAXX^Flox/WT^* and *DAXX^Flox/Flox^* cortical neurons infected with CRE lentivirus. Cells were left untreated or were treated with 50 mM KCl for 3 hr. ChIP with nonspecific rabbit IgG was used as background control. Data are mean ± SEM; n = 3–4; n.s., not significant; ^∗^p < 0.05; ^∗∗^p < 0.01; two-way ANOVA test with Bonferroni posttest.

DAXX has been recently implicated in loading of the histone variant H3.3 as part of a chaperone complex containing ATRX (Elsaesser and Allis, 2010). In view of the presence of both proteins at regulatory regions of selected IEGs, we speculated that DAXX could promote H3.3 loading at these loci. No data was available on induction of H3.3 deposition upon neuronal activation and the potential chaperones involved. To test this hypothesis, we first studied whether DAXX and H3.3 interact in neurons. Coimmunoprecipitation experiments showed that yellow fluorescent protein (YFP)-H3.3 pulled down endogenous DAXX (Figure 3A). Based on these data, we analyzed H3.3 association with regulatory regions of activity-regulated genes by using an H3.3-specific antibody (Figures S3A and S3B). *DAXX^Flox/WT^* or *DAXX^Flox/Flox^* neurons infected with CRE particles were depolarized with KCl for 3 hr. We found that neuronal activation clearly induced H3.3 deposition at regulatory regions of all genes included in this study (*Bdnf Exon IV*, *c-Fos*, *Npas4*, *Zif 268*, *Nurr1*, *Ier2*, *Gadd45g*, *Egr2*, *Dusp6*, and *Arc*; Figures 3B–3D and S3C). This was not due to increased nucleosome density, because anti-H4 ChIP failed to show increased H4 binding at regulatory regions of *Bdnf Exon IV* and *c-Fos* upon membrane depolarization (Figure S3D). DAXX depletion led to clear impairment in KCl-triggered loading of H3.3 at the regulatory elements of *Bdnf Exon IV* (Figure 3B; see regions 2 and 3 in Figure 2A), *c-Fos* (Figure 3C; see regions 1–3 in Figure 2B), *Egr2*, and *Dusp6* (Figure S3C). DAXX depletion did not interfere with nucleosome density at these genes (Figure S3D). Deposition of H3.3 at the *c-Fos* transcribed region was DAXX-independent, indicating that DAXX is not required for loading at this region (Figure 3C). This is in agreement with the HIRA-dependent deposition of H3.3 at actively transcribed genes (Goldberg et al., 2010). We observed residual induction of H3.3 loading at *Bdnf Exon IV*, *c-Fos*, *Egr2*, and *Dusp6* in DAXX-deficient cells, which, however, did not reach statistical significance for all regions analyzed (Figures 3B, 3C, and S3C). This trend was probably due to residual expression of DAXX in CRE-uninfected cells (Figures S2E and S2F). However, we cannot rule out a small degree of compensatory loading by other histone chaperones, such as CAF-1 (Drané et al., 2010). Finally, we analyzed whether H3.3 loading at genes not associated with DAXX was affected by DAXX loss. Both basal and activity-induced deposition of H3.3 at the regulatory elements of *Npas4*, *Zif 268*, *Nurr1*, *Ier2*, *Gadd45g*, and *Arc* remained unchanged in DAXX-deficient cells (Figures 3D and S3C). Thus, binding of DAXX to regulatory elements correlates with its ability to promote H3.3 loading.

### DAXX Regulates Activity-Dependent Transcription

**Figure 4:**
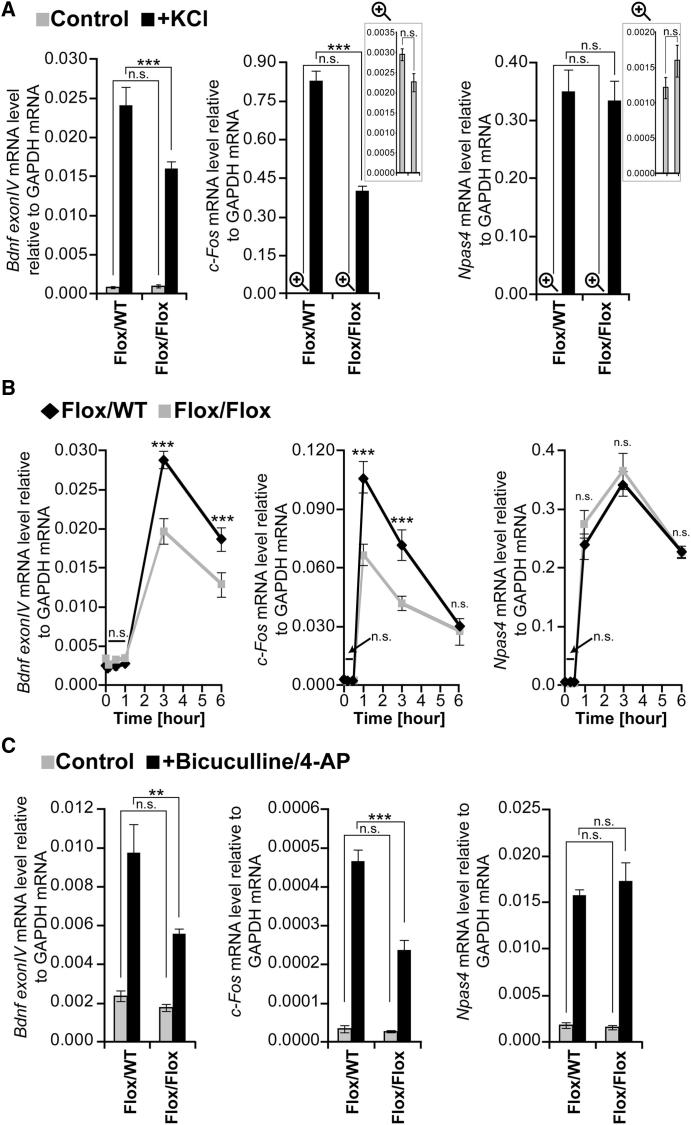
DAXX Regulates *Bdnf Exon IV* and *c-Fos* Transcriptional Induction (A) *DAXX^Flox/WT^* and *DAXX^Flox/Flox^* cortical neurons were infected with lentivirus encoding CRE recombinase. qPCR for *Bdnf Exon IV*, *c-Fos*, and *Npas4* expression was performed by using RNA extracted from 5 DIV neurons that were either untreated or membrane depolarized with 50 mM KCl for 3 hr. (B) *DAXX^Flox/WT^* and *DAXX^Flox/Flox^* cortical neurons were infected as above and treated with 50 mM KCl. Samples were collected at the indicated times during the time course and analyzed by qPCR. (C) *DAXX^Flox/WT^* and *DAXX^Flox/Flox^* cortical neurons were infected as above. Expression of *Bdnf Exon IV*, *c-Fos*, and *Npas4* was analyzed from 9 DIV neurons that were either untreated or treated with 50 μM bicuculline and 2.5 mM 4-AP for 3 hr. Data are mean ± SEM; n = 3; n.s., not significant; ^∗∗^p < 0.01; two-way ANOVA test with Bonferroni posttest.

We next sought to understand whether DAXX could regulate activity-dependent transcription at loci where it promotes H3.3 loading. H3.3 loading and transcription are tightly interconnected. Chromatin relaxation at actively transcribed genes has been proposed to promote H3.3 loading (Henikoff, 2008). In turn, reduced H3.3 loading is associated with impaired transcription at the *MyoD* locus (Yang et al., 2011). Moreover, H3.3 loading has been linked with activity-dependent transcription in myoblasts, T cells, and fibroblasts (Sutcliffe et al., 2009; Tamura et al., 2009; Yang et al., 2011). Finally, DAXX- and ATRX-mediated H3.3 loading has been implicated in regulation of pericentric and telomeric DNA repeat transcription (Drané et al., 2010; Goldberg et al., 2010). First, we analyzed whether transcriptional induction is required for H3.3 loading. As shown in Figure S4A, inhibition of Pol II completely abrogated activity-induced H3.3 loading at the promoter region of *Bdnf Exon IV*, as well as at enhancers, promoter, and gene body of *c-Fos*. We then tested the effect of DAXX loss on IEG induction. Although abrogation of DAXX did not affect basal mRNA levels, it led to a significant decrease in KCl-dependent induction of *Bdnf Exon IV*, *c-Fos*, *Egr2*, and *Dusp6* (Figures 4A and S4B). In contrast, *Npas4*, *Zif 268*, *Nurr1*, *Ier2*, *Gadd45g*, and *Arc* induction was not affected (Figures 4A and S4B). Decreased levels of *c-Fos* and *Bdnf Exon IV* in DAXX-deficient cells were not due to a delayed induction peak, because we detected a reduced amplitude of *Bdnf Exon IV* and *c-Fos* expression at all time points analyzed (Figure 4B). *Npas4* levels were not affected throughout the time course (Figure 4B).

We next tested the effect of DAXX loss in 9 DIV neurons treated with the GABA_A_ antagonist bicuculline, a more physiologically relevant stimulus. Similar to KCl, DAXX depletion led to decreased bicuculline-dependent induction of *Bdnf Exon IV*, *c-Fos*, *Egr2*, and *Dusp6*, whereas it did not affect the other genes included in the panel (Figures 4C and S4C).

Although these data suggest a link between DAXX-mediated histone loading and transcriptional induction of IEGs, DAXX role in transcriptional regulation could be independent of its histone loading function. In this respect, DAXX can associate with histone acetyl transferases, histone deacetylases, and DNA methyl transferases (Hollenbach et al., 2002; Kuo et al., 2005; Puto and Reed, 2008), thus suggesting that it could regulate transcription via modulation of histone acetylation and/or DNA methylation. To test this, we analyzed histone 3 (H3) and 4 (H4) acetylation at *Bdnf Exon IV* and *c-Fos* regulatory regions and methylation of CpG islands at the *Bdnf Exon IV* promoter. DAXX loss did not affect histone acetylation or CpG island methylation (Figures S4D–S4F). Taken together, these data suggest that DAXX-dependent regulation of H3.3 loading and activity-dependent transcription may be linked.

### DAXX Phosphorylation Is Regulated by Neuronal Activation

**Figure 5:**
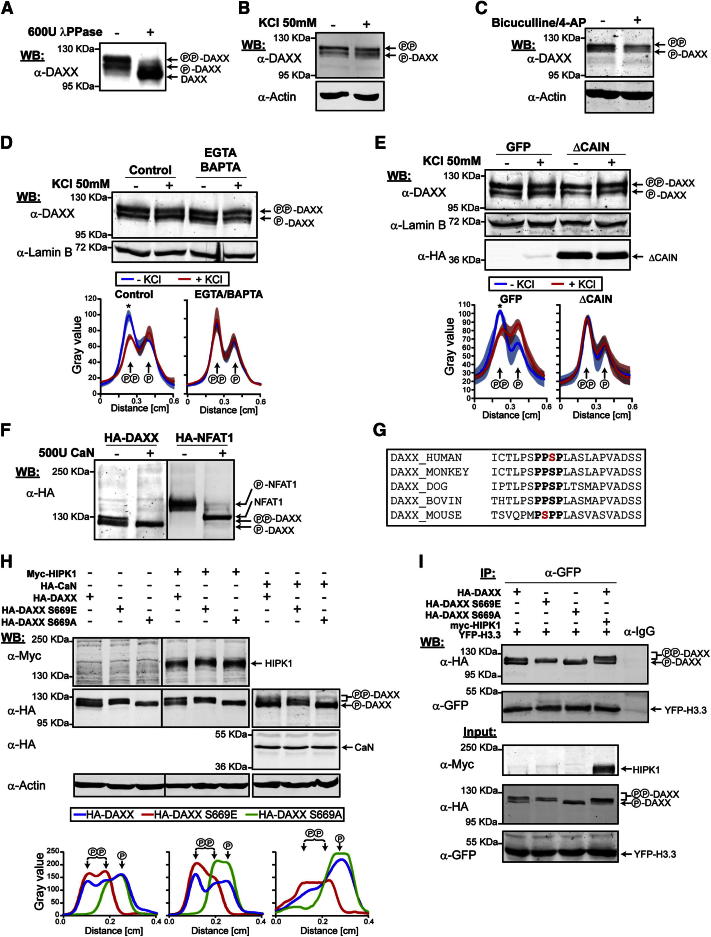
DAXX Phosphorylation Is Regulated by Calcineurin (A) Lysates from 5 DIV cortical neurons were left untreated or were treated with λ-phosphatase, followed by western blot analysis using α-DAXX antibody. PP-DAXX indicates the hyperphosphorylated band, whereas P-DAXX indicates intermediate phosphorylation, and DAXX indicates the fully unphosphorylated band. (B) Western blot from 5 DIV cortical neurons that were left untreated or were treated for 3 hr with 50 mM KCl. (C) Western blot from 9 DIV cortical neurons that were left untreated or were treated for 3 hr with 50 μM bicuculline and 2.5 mM 4-AP. (D) Top: whole-cell lysates were prepared from 5 DIV cortical neurons cultured in the absence or presence of EGTA/BAPTA (1 hr) to chelate extracellular and intracellular calcium, respectively. Extracts were probed with α-DAXX antibody. Bottom: the graphs represent image analysis of band intensity. (E) Same as in (D), but whole-cell lysates were prepared from 5 DIV cortical neurons infected with lentivirus expressing GFP or the calcineurin inhibitor ΔCAIN. (F) HA-DAXX and HA-NFAT1 were immunoprecipitated with α-HA antibody from transfected 293T cells. Immunoprecipitates were treated with purified calcineurin (+CaN) or were mock treated (−CaN) and followed by western blot analysis with α-HA antibody. P-NFAT1 and NFAT1 indicate hyperphosphorylated and unphosphorylated proteins, respectively. (G) Conservation of the protein sequence (bold) around the serine known to be phosphorylated by HIPK1 (red) among mammalian species. (H) α-HA western blotting of lysates from 293T cells transfected with different combinations of HA-DAXX (wild-type), HA-DAXX S669E (phosphomimetic), HA-DAXX S669A (phosphomutant), Myc-HIPK1, HA-CaN (constitutively active form of calcineurin), and empty vector. The bottom graphs represent image analysis of band intensities. (I) Interaction between H3.3 and HA-DAXX (wild-type), HA-DAXX S669E (phosphomimetic), and HA-DAXX S669A (phosphomutant) in 293T cells. Extracts from 293T cells transfected with different combinations of HA-DAXX (wild-type), HA-DAXX S669E (phosphomimetic), HA-DAXX S669A (phosphomutant), Myc-HIPK1, YFP-H3.3, and empty vector were immunoprecipitated with anti-GFP or control antibody. The immunoprecipitates were analyzed by western blotting using the indicated antibodies.

We next investigated whether DAXX is regulated upon neuronal activation. In this respect, neuronal activation promotes changes in the phosphorylation status of essential regulators of activity-dependent transcription, such as CREB, MEF2, NFAT, and MeCP2 (Cohen and Greenberg, 2008). DAXX is known to be phosphorylated at several residues (Chang et al., 2011; Ecsedy et al., 2003), leading to differential migration in SDS-PAGE (Ecsedy et al., 2003). We detected similar DAXX forms in extracts from cultured cortical neurons, which were abolished by treatment with λ-phosphatase (Figure 5A). KCl or bicuculline treatment led to downregulation of hyperphosphorylated DAXX (Figures 5B and 5C). These changes were calcium dependent, because pretreatment with the extracellular and intracellular chelators EGTA and BAPTA abrogated this effect (Figure 5D).

Calcineurin, a key phosphatase involved in calcium-dependent signaling cascades, dephosphorylates key transcription factors in neurons, such as MEF2 and NFAT (Flavell et al., 2006; Graef et al., 1999; Shalizi et al., 2006). To test whether the modulation of DAXX phosphorylation was calcineurin-dependent, we infected cortical neurons with lentiviral particles encoding a calcineurin inhibitory peptide (ΔCAIN; Lai et al., 1998). ΔCAIN prevented the modulation of DAXX phosphorylation upon membrane depolarization (Figure 5E). Furthermore, DAXX was dephosphorylated in a calcineurin-dependent manner in 11 DIV cortical neurons exposed to glutamate (Figure S5A). Finally, recombinant calcineurin dephosphorylated DAXX in vitro, showing that DAXX was a direct substrate (Figure 5F). Taken together, these findings indicate that DAXX phosphorylation status is regulated by calcium and calcineurin in neurons.

As DAXX did not undergo complete dephosphorylation upon neuronal activation, it is conceivable that specific residues may be targeted. In this respect, DAXX has been shown to be phosphorylated at the conserved serine 669 (S669) (Figure 5G) by the homeodomain-interacting protein kinase 1 (HIPK1) (Ecsedy et al., 2003). S669 phosphorylation has been previously shown responsible for the appearance of slow-migrating DAXX forms in SDS-PAGE (Ecsedy et al., 2003). We generated hemagglutinin (HA)-DAXX constructs expressing nonphosphorylatable (S669A) and phosphomimetic (S669E) DAXX mutants. Whereas S669E DAXX migrated like hyperphosphorylated DAXX, migration of the S669A mutant corresponded to hypophosphorylated DAXX (Figure 5H). Overexpression of an active form of calcineurin led to reduced migration of wild-type (WT) DAXX but did not affect the two mutants (Figure 5H). Similarly, coexpression of HIPK1 promoted hyperphosphorylation of WT DAXX only (Figure 5H). These results indicate that DAXX S669 phosphorylation is modulated by calcineurin.

We next explored whether the phosphorylation status of DAXX regulates its interaction with H3.3 and ATRX. As shown in Figure 3A, we found an enrichment of endogenous hypophosphorylated DAXX in YFP-H3.3 immunoprecipitates in neurons. Similar findings were obtained with exogenously expressed WT DAXX in 293T cells (Figure 5I) as well as in neurons (Figure S5B). HIPK1 overexpression led to DAXX hyperphosphorylation, but only a small proportion of hyperphosphorylated DAXX was found to be associated with H3.3 (Figure 5I). This enrichment did not appear due to reduced H3.3 affinity for hyperphosphorylated DAXX, because similar levels of S669E and S669A mutants were found to be associated with H3.3 (Figure 5I). Finally, we failed to detect any effect of DAXX phosphorylation status on its ability to interact with ATRX (Figure S5C).

### Calcineurin-Dependent Dephosphorylation of Serine 669 Regulates DAXX Function upon Neuronal Activation

**Figure 6:**
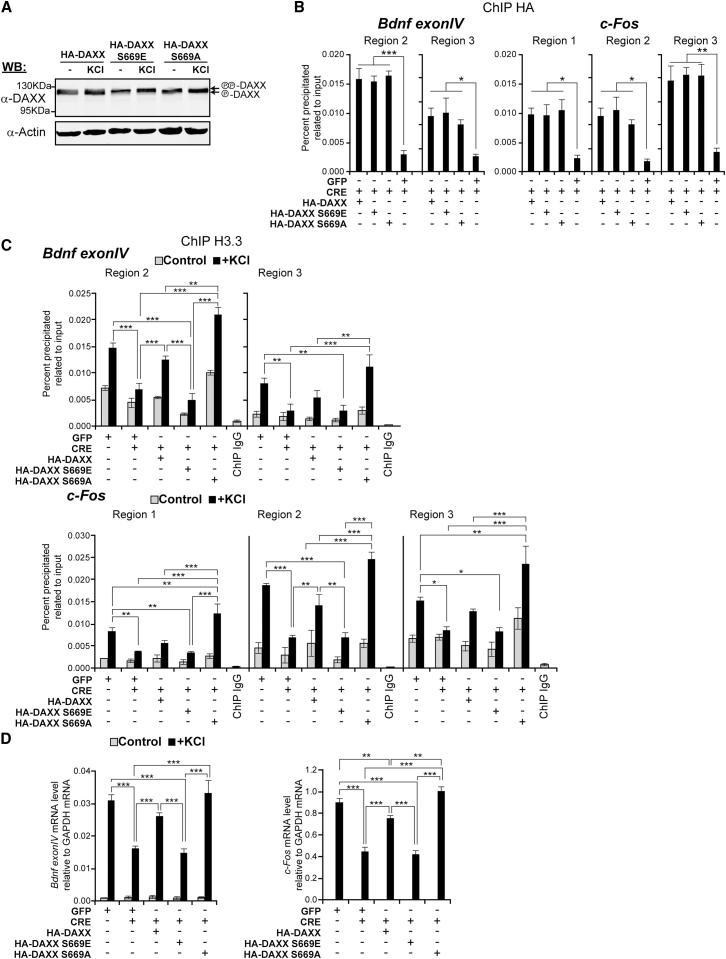
DAXX Phosphorylation Status Regulates Deposition of H3.3 and Immediate Early Gene Expression (A) Whole-cell lysates were prepared from 5 DIV *DAXX^Flox/Flox^* cortical neurons that were left untreated or were treated for 3 hr with 50 mM KCl. Cells were infected with WT HA-DAXX, S669E HA-DAXX, and S669A HA-DAXX lentiviral constructs. (B) ChIP analysis of recombinant HA-tagged DAXX constructs enrichment at the *Bdnf Exon IV* and *c-Fos* regulatory regions. *DAXX^Flox/Flox^* cortical neurons were infected with a combination of CRE/GFP or CRE/DAXX vectors (CRE/GFP, DAXX; multiplicity of infection [moi] 0.75/1.00). Regions were selected based on data presented in Figure 2. We performed ChIP by using CRE/GFP-infected *DAXX^Flox/Flox^* cells as background control. Data are mean ± SEM from n = 3; only statistically significant differences are indicated; ^∗^p < 0.05; ^∗∗^p < 0.01; ^∗∗∗^p < 0.001; two-way ANOVA test with Bonferroni posttest. (C) ChIP analysis of H3.3 enrichment at the *Bdnf Exon IV* and *c-Fos* regulatory regions. *DAXX^Flox/Flox^* cortical neurons were infected with a GFP lentiviral vector (moi 1.75) or with a combination of CRE/DAXX vectors (CRE/GFP, moi 0.75/1.00; CRE/HA-DAXX constructs, moi 0.75/1.00). Cells were either left untreated or membrane depolarized with 50 mM KCl for 3 hr. Regions were selected based on data presented in Figure 2. ChIP with nonspecific rabbit IgG was used as background control. Data are mean ± SEM from n = 3; n.s., not significant; ^∗∗^p < 0.01; ^∗^p < 0.05; ^∗∗∗^p < 0.001; two-way ANOVA test with Bonferroni posttest. (D) *DAXX^Flox/Flox^* cortical neurons were infected as in (C). qPCR analysis of *Bdnf Exon IV* and *c-Fos* expression was performed by using RNA from infected 5 DIV cells that were either untreated or were membrane depolarized with 50 mM KCl for 3 hr. Data are mean ± SEM from n = 3; only statistically significant differences are indicated; ^∗∗^p < 0.01; ^∗∗∗^p < 0.001; two-way ANOVA test with Bonferroni posttest.

Because DAXX/H3.3 complexes are enriched in hypophosphorylated DAXX, we reasoned that DAXX phosphorylation status could play a role in the regulation of H3.3 deposition. To test this hypothesis, we performed rescue experiments in *DAXX^Flox/Flox^* neurons. CRE promoted efficient deletion of endogenous *DAXX* in cells coinfected either with a green fluorescent protein (GFP) vector or DAXX constructs (Figures S6A–S6C). Similar expression levels of WT, S669A, and S669E DAXX were achieved in transduced neurons (Figure 6A). Upon membrane depolarization, migration of S669A and S669E DAXX mutants was not affected, whereas levels of hyperphosphorylated WT DAXX decreased (Figure 6A). Furthermore, no significant differences in association with *Bdnf Exon IV* and *c-Fos* regulatory regions were detected in between the constructs both at steady state and upon KCl treatment (Figure 6B). As expected, WT DAXX rescued H3.3 loading at *Bdnf Exon IV* and *c-Fos* regulatory regions in CRE-infected *DAXX^Flox/Flox^* neurons (Figure 6C). Notably, S669A DAXX had a more pronounced rescuing activity at most regions analyzed (Figure 6C). Conversely, S669E DAXX failed to rescue loading at all regions (Figure 6C).

We then tested whether DAXX phosphorylation also affected its ability to regulate transcription. WT and S669A DAXX rescued expression of *Bdnf Exon IV* and *c-Fos*. In contrast, S669E DAXX was impaired in this function (Figure 6D). Notably, S669A DAXX was more potent in rescuing *c-Fos* induction compared to WT DAXX (Figure 6D). Taken together, these data suggest that calcium-dependent dephosphorylation of DAXX positively affects H3.3 loading and transcriptional regulation.

## Discussion

Although considerable progress has been made in our understanding of activity-dependent chromatin remodeling in neurons, this process is far from being fully elucidated. In the present study, we implicate loading of the histone variant H3.3 as part of activity-triggered chromatin changes in neurons. In particular, we show that the histone chaperone DAXX regulates activity-dependent H3.3 deposition and transcription through a mechanism involving a calcium-dependent phosphorylation switch.

DAXX interacts with PML and ATRX, known regulators of brain development (Bérubé et al., 2005; Gibbons et al., 1995; Regad et al., 2009). Differentiated cortical neurons coexpress DAXX and ATRX, which are found in the nucleoplasm and are associated with heterochromatic foci, phenocopying the distribution of ATRX-binding protein MeCP2 (Martinowich et al., 2003). Furthermore, DAXX and ATRX interact in whole-brain extracts and isolated neurons. Both ATRX and MeCP2 are involved in chromatin remodeling and transcriptional control (Guy et al., 2011; Xue et al., 2003). In particular, MeCP2 has been shown to regulate transcriptional activation of the immediate early gene *Bdnf Exon IV* upon enhanced neuronal activity (Chen et al., 2003a; Martinowich et al., 2003). Our data show that DAXX associates with the same regulatory region of the *Bdnf Exon IV* promoter occupied by MeCP2. In addition, it is also present at regulatory regions of the IEGs *c-Fos*, *Egr2*, and *Dusp6*. In contrast, it is absent from *Npas4*, *Zif 268*, *Nurr1*, *Ier2*, *Gadd45g*, and *Arc* regulatory elements. This raises the question of how gene-specific localization of DAXX is regulated. A candidate for this function is ATRX. DAXX and ATRX interact in neurons and bind the same IEG regulatory regions. Furthermore, ATRX has been recently shown to recognize specific histone tail modifications and DNA conformation (Eustermann et al., 2011; Iwase et al., 2011), thus suggesting that these marks could confer specificity to DAXX binding.

DAXX is a chaperone for the histone variant H3.3, which, unlike H3, is transcribed in a replication-independent manner. Because neurons do not proliferate, H3.3 is the predominant H3 variant expressed in neurons, exemplified by the increased ratio of H3.3/H3 in the mouse brain during postnatal development (Piña and Suau, 1987). So far, regulation of H3.3 loading in neurons has not been studied. Our data show that DAXX interacts with H3.3 in neurons, thus suggesting that it may regulate its deposition at activity-regulated genes. Indeed, we demonstrate that H3.3 is loaded onto IEG regulatory regions upon membrane depolarization. H3.3 loading was dependent on active transcription, as inhibition of Pol II blocked its deposition, thus suggesting that initiation of transcription is essential for histone variant deposition. It has to be noted that the loading of H3.3 at enhancer regions could be explained by the presence of actively transcribing Pol II (Kim et al., 2010). Although the histone chaperone HIRA is responsible for H3.3 loading at TSS and bodies of active genes (Goldberg et al., 2010), the chaperone controlling H3.3 deposition at gene regulatory regions was not known. We discovered that H3.3 deposition at regulatory elements of selected IEGs (*Bdnf Exon IV*, *c-Fos*, *Egr2*, and *Dusp6*) mainly relies on DAXX. These data suggest that DAXX is one of the previously unknown chaperones controlling H3.3 loading at regulatory elements. Because downregulation of DAXX does not change H3.3 loading at regulatory elements of *Npas4*, *Zif 268*, *Nurr1*, *Ier2*, *Gadd45g*, and *Arc*, further studies are needed to discover the chaperone responsible for this activity. Potential candidates are DEK and HIRA (Elsaesser and Allis, 2010; Sawatsubashi et al., 2010).

Our findings show that impaired H3.3 loading in DAXX-depleted cells correlates with reduced transcriptional induction by neuronal depolarization. Similar findings were obtained by activating neurons through the use of the GABA_A_ antagonist bicuculline. These results show that there is correlation between the presence of DAXX at specific regulatory elements, DAXX-dependent H3.3 loading at these same regions, and transcriptional induction. The effect on transcriptional regulation could be independent of DAXX chaperone function. In this respect, DAXX has been reported to regulate histone acetylation as well as DNA methylation (Kuo et al., 2005; Puto and Reed, 2008). However, no changes in H3 and H4 acetylation or CpG island methylation were observed in DAXX-deficient neurons. This raises the question of whether activity-regulated H3.3 deposition at regulatory regions could regulate gene transcription. This remains an unanswered question in the epigenetics field, due to the interdependent relationship between transcription, histone eviction, and de novo loading processes. Loss of histone chaperones has been shown to affect transcription (Placek et al., 2009; Tamura et al., 2009; Yang et al., 2011). For instance, loss of HIRA impairs both H3.3 loading and transcription of the *MyoD* gene (Yang et al., 2011). Furthermore, H3.3 loading at telomeres and pericentric heterochromatin, which is dependent on DAXX and ATRX, have been suggested to modulate transcription of respective DNA repeats (Drané et al., 2010; Goldberg et al., 2010). Vice versa, H3.3 overexpression leads to changes in transcription of selected genes (Jin and Felsenfeld, 2006). However, in these studies, nonspecific effects of global changes downstream which altered loading could not be excluded. Notably, a recent study has shown that mutations of H3.3 found in glioma are associated with specific alterations of gene expression (Schwartzentruber et al., 2012; Wu et al., 2012), thus suggesting that changes in H3.3 deposition may affect gene transcription and potentially contribute to disease pathogenesis. Mechanistically, it has been suggested that incorporation of histone variants can lead to nucleosome destabilization. In this respect, ASF1-mediated loading may affect transcription in yeast because of the destabilizing effect of histone variants on nucleosomes, which in turn would favor their more rapid and efficient eviction by Pol II (Schwabish and Struhl, 2006). In mammalian cells, variant nucleosomes containing H3.3/H2AZ are unstable, thus suggesting a more accessible state of chromatin marked by these nucleosome variants (Jin et al., 2009). It is possible that DAXX could promote loading of H3.3/H2AZ-containing nucleosomes at regulatory elements of activity-regulated genes, thus making them more easily displaceable. Finally, it is also possible that H3.3 deposition could have more long-lasting effects on transcriptional regulation. In this respect, it has been also implicated in controlling epigenetic memory and maintenance of active transcriptional state (Ng and Gurdon, 2008). Therefore, loss of DAXX-dependent H3.3 loading could also regulate long-lasting chromatin regulation of IEGs.

DAXX association with regulatory elements is not affected by neuronal activity. Instead, neuronal activation leads to decreased DAXX phosphorylation. We demonstrate that DAXX phosphorylation is regulated by calcineurin, a key calcium-dependent phosphatase involved in dephosphorylation of MEF2 and NFAT (Flavell et al., 2006; Graef et al., 1999; Shalizi et al., 2006). Calcineurin dephosphorylates DAXX at the serine 669, which is under the control of HIPKs (Ecsedy et al., 2003). Interestingly, HIPK2 is known to regulate transcription in neurons (Wiggins et al., 2004). In resting neurons, HIPK2 phosphorylates MecP2 at serine 80 (Bracaglia et al., 2009), contributing to transcriptional repression (Tao et al., 2009). Thus, it is conceivable that interplay between HIPKs and calcineurin could be an important regulatory node for regulation of chromatin remodeling and transcription in neurons. We investigated whether DAXX phosphorylation status could affect its ability to promote H3.3 deposition and transcription. The phosphomimetic S669E DAXX mutant is unable to promote either H3.3 loading or transcription in rescue experiments. In contrast, the S669A mutant rescues both H3.3 loading and transcription in DAXX-deficient cells. Notably, the effect of S669A DAXX on H3.3 loading is greater than WT DAXX. It is worth noting that Cabin/CAIN, a negative regulator of calcineurin (Lai et al., 1998), is a component of the HIRA complex (Ray-Gallet et al., 2011; Tagami et al., 2004), thus suggesting that other H3.3 chaperone complexes may be regulated in a calcium- and calcineurin-dependent manner.

Would DAXX phosphorylation affect its interaction with H3.3? We found an enrichment of hypophosphorylated DAXX in H3.3 immunoprecipitates. Overexpression of the S669 kinase HIPK1 only led to a small increase in the amount of hyperphosphorylated DAXX in H3.3 pull-downs. This is unlikely due to increased affinity of hypophosphorylated DAXX for H3.3, because similar levels of S669E and S669A mutants were found in H3.3 pull-downs. Considering the loss-of-function property of S669E DAXX in rescue experiments, it is conceivable that dephosphorylation of DAXX when in complex with H3.3 could be required for its chaperone activity. Therefore, the functional impairment of the S669E mutant could be due to lack of dephosphorylation rather than reduced binding.

Taken together, these findings implicate DAXX in the regulation of histone variant loading and transcription in the central nervous system. In particular, we propose a model by which activity-induced calcium signaling promotes transcriptional initiation as well as DAXX dephosphorylation. Both events are key for stimulation of DAXX-dependent H3.3 loading. Because DAXX loss impairs not only H3.3 loading, but also induction of activity-regulated genes, it is possible that H3.3 deposition could underlie aspects of stimulus-inducible gene transcription. More broadly, our work raises the prospect that dynamic replacement of histone variants could play an important role in genome remodeling and transcriptional regulation in the nervous system.

## Experimental Procedures

See Supplemental Experimental Procedures.

### Plasmids

N-terminal HA-tagged mouse DAXX and derivatives were cloned into pcDNA3.1 (Invitrogen) or pCMS-EGFP for transfection or into TRIP-PGK-ATGm-MCS-WHV (D. Trono's laboratory, see Acknowledgments) for lentivirus production. DAXX phosphomimetic (S669E) and phosphomutant (S669A) were generated by PCR mutagenesis as described previously (Nelson and Long, 1989). Plasmids were controlled by sequencing. Plasmids expressing the calcineurin inhibitor ΔCAIN (Lai et al., 1998) and the constitutively active calcineurin (O'Keefe et al., 1992) were a gift from A. Genazzani. Each construct was subcloned into TRIP-PGK-ATGm-MCS-WHV for lentivirus production. The plasmid for the expression of HIPK1 was a gift from P. Leder (Harvard University). Plasmids used for lentivirus production (pMD.G and pCMV delta R8.91) were from D. Trono's laboratory. YFP-H3 and YFP-H3.3 plasmids are from Addgene (Addgene plasmids 8694 and 8693); YFP-H3.3 sequence was subcloned into TRIP-PGK-ATGm-MCS-WHV for lentivirus production.

### Production of Conditional DAXX Knockout Mouse

The *DAXX^Flox/Flox^* mouse line was obtained from P. Leder. Details can be found on the Jackson Laboratories webpage. The targeting vector contained a *neomycin* (PGKneo) gene surrounded by flipase sequences (FRT), which were removed in embryonic stem cells. *DAXX Exon II* sequence was flanked by LoxP sites. All mice were maintained in the 129S background. Mice were bred and subjected to listed procedures under the Project License 80-2325, released from the Home Office, UK. Genotyping of mice was performed by using Extract-N-Amp Tissue PCR Kit (Sigma-Aldrich) with primers inside exon I (5′-AGCAGTAACTCCGGTAGTAGGAAG) and exon II (5′-AGGAACGGAACCACCTCAG). To check the recombination induced by the CRE recombinase, we added an additional primer inside exon III (5′-GAAGGCGGCGAGCCAATGTG). An alternative primer inside the 5′ UTR of exon I (5′-CCCTCAGGGGAATTTGAACC) was used in Figure S5.

### Culture of Cortical Neurons

Cortical neurons were prepared from mouse embryonic day 16 (E16) cerebral cortices. The cortices were dissociated into single-cell suspension by trypsin digestion and mechanical trituration. The triturated cells were passed through a 40 μm cell strainer. Cells were first cultured in Neurobasal Medium (Invitrogen) supplemented with 10% fetal bovine serum (Invitrogen), 2 mM glutamine (Invitrogen), 100 U/ml penicillin, and 100 μg/ml streptomycin (Invitrogen) for 1 hr; then the medium was replaced with culture medium (Neurobasal Medium, B27, Invitrogen), 2 mM glutamine, 100 U/ml penicillin, and 100 μg/ml streptomycin). Cells were plated at 8 × 10^5^ cells/ml in 6-well plates previously coated with poly-D-lysine (Sigma-Aldrich). Neuronal cultures were treated overnight in 1 μM tetrodotoxin (Tocris) to reduce endogenous neuronal activity prior to stimulation. Neuron depolarization was induced by adding 50 mM KCl to the medium for the indicated times. For neurons kept in culture until 9 DIV, cells were treated with 10 μM Ara-C (Sigma C6645) at 4 DIV, and half the medium was replaced with fresh medium 2 days after 5 DIV. Neurons were treated with 50 μM bicuculline (Sigma B7561) and 2.5 mM 4-AP (Sigma A78403) for the indicated times.

### Chromatin Immunoprecipitation

We cultured 8 × 10^6^ cortical neurons in 10 cm petri dishes for 5 DIV. For chromatin immunoprecipitation (ChIP), the ChIP Assay Kit (Millipore) was used according to the manufacturer's instructions. Briefly, cells were crosslinked in 1% formaldehyde, lysed in SDS buffer, and sonicated. Immunoprecipitation was performed overnight with the relevant antibody: DAXX (Santa Cruz Biotechnology sc-7152), ATRX (Santa Cruz Biotechnology sc-15408), MeCP2 (Millipore 07-013), H3.3 (Abcam ab62642), H4 (Millipore 17-10047), acH3 (Millipore 06-599), acH4 (Millipore 06-866), HA (Abcam ab9110), or rabbit IgG (Cell Signaling 2729). The precipitated protein-DNA complexes were eluted from the antibody with 1% SDS and 0.1 M NaHCO3, and then incubated at 65°C overnight in 200 mM NaCl to reverse formaldehyde crosslinks. After proteinase K and RNase digestion, DNA was purified with the MinElute PCR Purification Kit (QIAGEN). Input samples represent 1% of total chromatin input. For quantitative ChIP, amplification was performed with Maxima SYBR Green qPCR Master Mix (Fermentas). Percent input was calculated with the formula 100 × 2^∧^(Ct_adjusted input_ − Ct_IP_). Input DNA Ct was adjusted from 1% to 100% equivalent by subtracting 6.644 Cts (Log_2_100) from original Ct_input_. Primers sequences are in Table S1.

### Analysis of ChIP-Seq Data

Analysis was performed with the UCSC Genome Browser by using published data given in Table S6 of Kim et al. (2010).

### Western Blot

Established methods were used for western blotting. Additional details can be found in the Supplemental Experimental Procedures.

### Coimmunoprecipitation

Coimmunoprecipitation experiments were conducted by using extracts from primary neurons and 293T cells. Additional details can be found in the Supplemental Experimental Procedures.

### RNA Isolation, RT-PCR, and Quantitative Real-Time PCR Analysis

Total RNA was prepared from primary neurons. Additional details can be found in the Supplemental Experimental Procedures. Primer sequences are in Table S1.

### In Vitro Phosphatase Assay

Phosphatase assays were conducted by using purified calcineurin. Additional details can be found in the Supplemental Experimental Procedures.

### Virus Preparation and Infection

Lentiviral supernatants were prepared as described previously (Salmon and Trono, 2006). Additional details can be found in the Supplemental Experimental Procedures.

### Immunohistochemistry

Immunohistochemistry was performed on tissue sections from mouse brain. Additional details can be found in the Supplemental Experimental Procedures.

### Immunofluorescence

Details of immunofluorescence techniques can be found in the Supplemental Experimental Procedures.

### Image Analysis

Western blot scans were analyzed by using ImageJ. A rectangle was drawn around the band, and analysis was done by using the Plot Profile command. Plot Profile command displays, for a rectangular selection, a “column average plot,” in which the x axis represents the horizontal distance through the selection and the y axis indicates the vertically averaged pixel intensity.

### Statistical Analysis

Mean values are presented with error bars corresponding to ±SEM. Statistical analysis was performed by using Prism statistical analysis software (GraphPad). Significance is indicated as ^∗∗∗^p < 0.001; ^∗∗^p < 0.01; ^∗^p < 0.05.

## Supplemental Information

Document S1. Figures S1–S6, Table S1, and Supplemental Experimental Procedures
