# Supplementary material for: Calcium-Dependent Dephosphorylation of the Histone Chaperone DAXX Regulates H3.3 Loading and Transcription upon Neuronal Activation
Source: Neuron. 2012 Apr 12;74(1):122–35. doi: 10.1016/j.neuron.2012.02.021 (PMC3657165; doi:10.1016/j.neuron.2012.02.021)
Supplement: Document S1. Figures S1–S6, Table S1, and Supplemental Experimental Procedures [file mmc1.pdf]

Neuron, Volume *74*

Supplemental Information

Calcium-Dependent Dephosphorylation of the Histone Chaperone DAXX Regulates H3.3 Loading and Transcription upon Neuronal Activation

David Michod, Stefano Bartesaghi, Amel Khelifi, Cristian Bellodi, Laura Berliocchi, Pierluigi Nicotera, and Paolo Salomoni

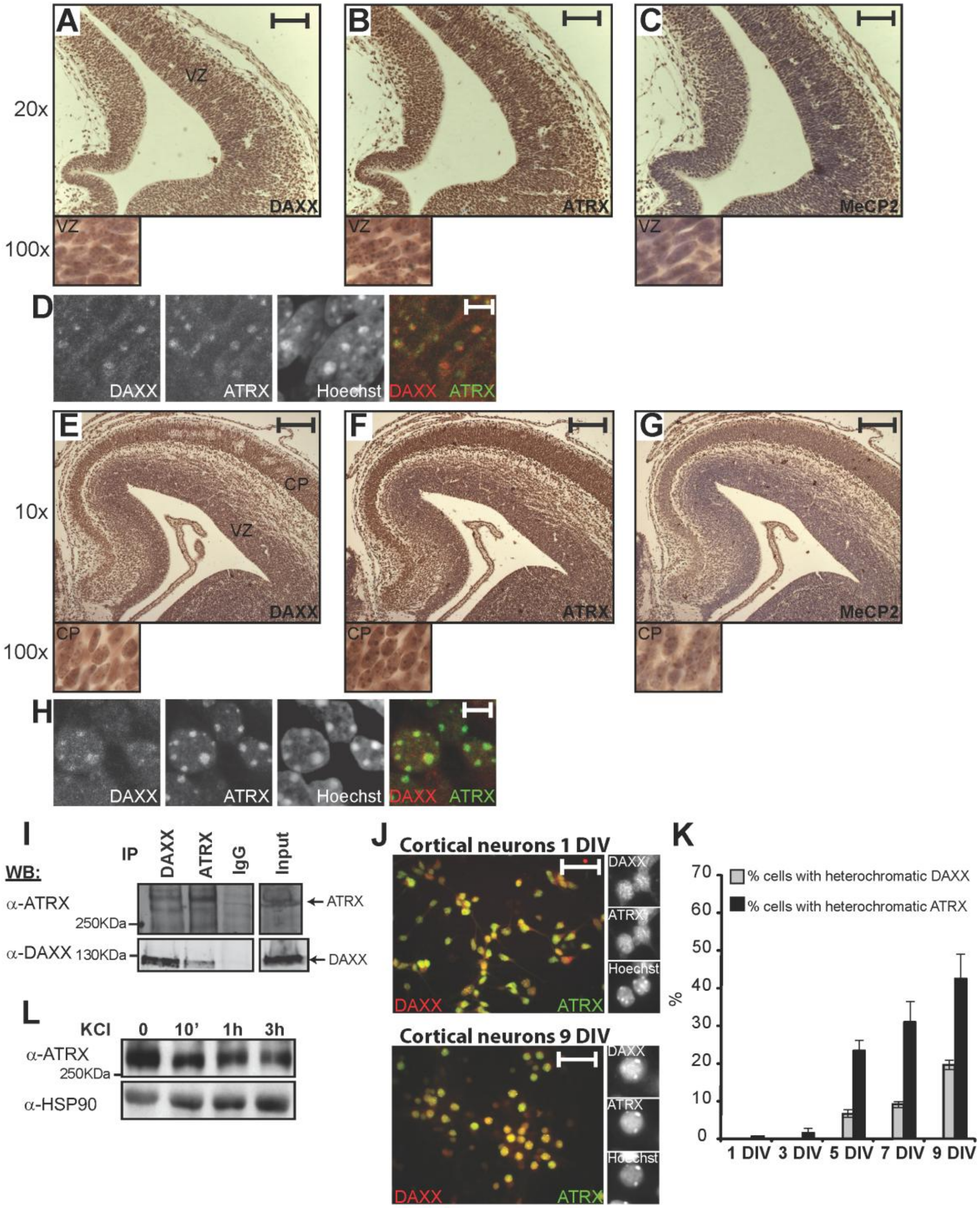

Supplementary Figure 1 (Michod & al.)

Figure S1 (related to Figure 1). DAXX is expressed in the mouse brain throughout development

(A-C) Immunohistochemistry of DAXX, ATRX and MeCP2 on coronal sections of mouse brain at embryonic day 12.5 (E12.5). Images show the cortical ventricular zone (VZ). Scale bars represent 100μm. (D) DAXX/ATRX immunofluorescence in VZ at E12.5. Scale bars represent 5μm. (E-G) Immunohistochemistry of DAXX, ATRX and MeCP2 on coronal section of mouse brain at E17.5. Scale bars represent 200μm. Images show DAXX and ATRX staining in the VZ. Scale bars represent 5μm. (H) DAXX/ATRX immunofluorescence in VZ at E17.5. Scale bars represent 5μm. (I) Interaction between DAXX and ATRX in mouse brain extracts. Extracts from mouse cortex at post-natal day 21 were immunoprecipitated (IP) with anti-DAXX, anti-ATRX or a control antibody. The immunoprecipitates were analyzed by western blotting using the indicated antibodies. (J) Immunofluorescence analysis of DAXX and ATRX localization in DIV1 and DIV9 cortical neurons. Scale bars represent 20μm. (K) Quantification of DAXX and ATRX colocalization in heterochromatin between DIV1-9. Data are mean ± SEM from n = 3. (L) ATRX expression in cortical neurons DIV5 upon 50mM KCl treatment for the indicated time.

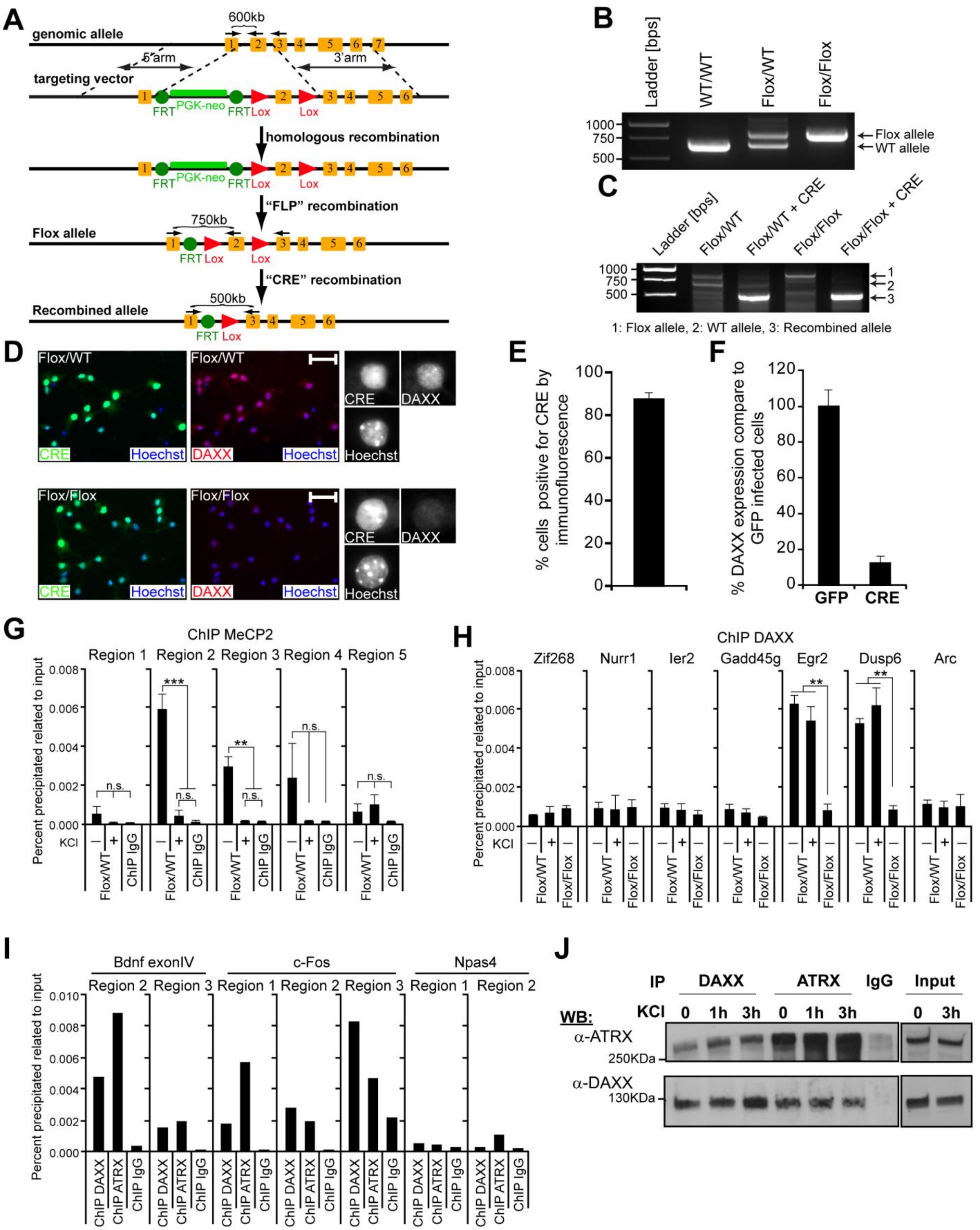

Supplementary Figure 2 (Michod & al.)

Figure S2 (related to Figure 2). DAXX$^{Flox/Flox}$ mice and analysis of MeCP2 and ATRX at regulatory elements of immediate early genes

(A) *DAXX* genomic locus, *DAXX* floxed gene targeting construct, *DAXX* floxed locus following homologous recombination are shown. Final *DAXX* locus following deletion of the FRT-flanked *Neomycin resistance* gene (*PGK-Neo*) and CRE-mediated deletion of *DAXX exon II* are also shown. *DAXX* exons are shown as orange boxes and are numbered. Arrows indicate location of primers used for the genotyping. (B) Genotyping of mice *DAXX*$^{WT/WT}$, *DAXX*$^{Flox/WT}$ *and DAXX*$^{Flox/Flox}$ using the primers indicated in panel (A). Amplification of WT and Flox alleles are indicated. (C) Genotyping of mice *DAXX*$^{WT/WT}$ and *DAXX*$^{Flox/Flox}$ infected with CRE recombinase particles using the primers indicated in panel A. Amplification of WT, Flox and recombined alleles are indicated. (D) Immunofluorescence of DAXX and CRE in cortical neurons DIV5 with a *DAXX*$^{Flox/WT}$ or *DAXX*$^{Flox/Flox}$ genotype. Cells were infected with lentivirus expressing the CRE recombinase. (E) Quantitative analysis of panel D. (F) qPCR analysis of DAXX expression in cortical neurons *DAXX*$^{Flox/Flox}$ infected with GFP or CRE particles. (G) ChIP analysis of MeCP2 enrichment at selected regions of *Bdnf exon IV* in *DAXX*$^{Flox/WT}$ cortical neurons prior infected with CRE lentivirus and left unstimulated or stimulated with 50mM KCl for 3 hours. ChIP with rabbit non-specific IgG was used as background control. (n = 3; n.s. non specific, $^{**}P<0.01$, $^{***}P<0.001$; Two-Way ANOVA test with Bonferroni post-test). (H) ChIP analysis of DAXX enrichment at promoter regions of selected IEGs in *DAXX*$^{Flox/WT}$ cortical neurons in the absence or presence of 50mM KCl (3 hours). We performed ChIP using CRE-infected *DAXX*$^{Flox/Flox}$ cells as background control. (n = 3; only statistically significant differences are indicated, $^{**}P<0.01$; Two-Way ANOVA test with Bonferroni post-test). (I) ChIP analysis of ATRX enrichment at regulatory regions of selected IEGs in cortical neurons DIV5. (J) Interaction between DAXX and ATRX in cortical neurons extracts. Extracts from cortical neurons DIV5 treated with 50mM KCl for the indicated time were immunoprecipitated (IP) with anti-DAXX, anti-ATRX or a control antibody. The immunoprecipitates were analyzed by western blotting using the indicated antibodies.

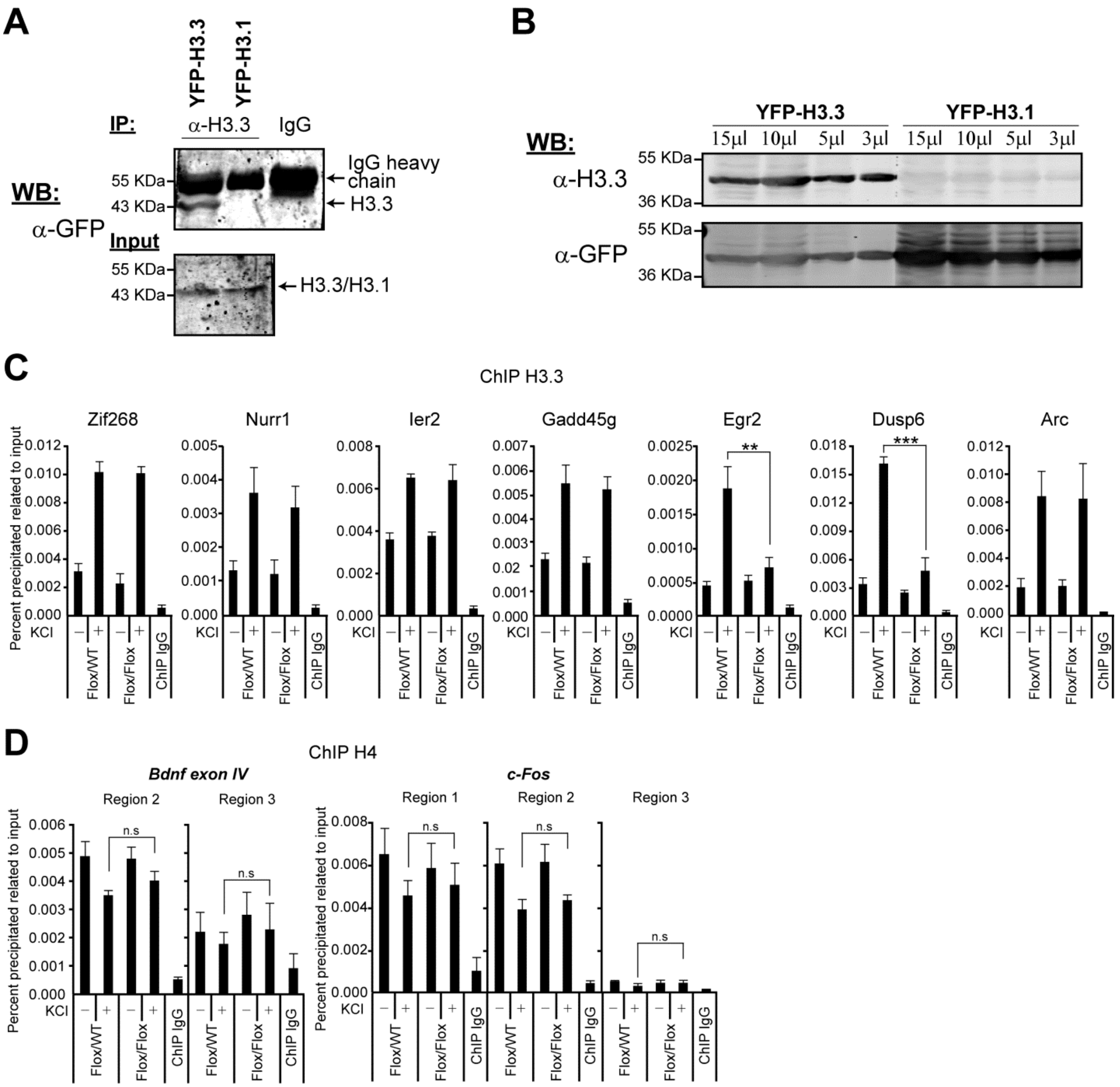

Supplementary Figure 3 (Michod & al.)

Figure S3 (related to Figure 3). DAXX regulates deposition of H3.3 at promoter regions of IEGs

(A and B) Specificity of H3.3 antibody used for ChIP analysis. (A) H3-YFP or H3.3-YFP were immunoprecipitated (IP) from 293T cell overexpressing these constructs with an anti GFP antibody The immunoprecipitates were analyzed by western blotting using the indicated antibody. (B) Western-blot analysis of extracts from 293T cells overexpressing H3-YFP or H3.3-YFP. Volumes of the extracts loaded are indicated. (C) ChIP analysis of H3.3 enrichment at promoter region of genes analyzed in Figure S2H. ChIP was performed using chromatin from $DAXX^{Flox/WT}$ and $DAXX^{Flox/Flox}$ cortical neurons infected with CRE lentivirus. Cells were left untreated or treated with 50mM KCl for 3 hours. ChIP with rabbit non-specific IgG was used as background control. Data are mean ± SEM (n = 3; $^{**}P<0.01$, $^{***}P<0.001$; Two-Way ANOVA test with Bonferroni post-test). (D) ChIP analysis of H4 enrichment at the regulatory regions of *Bdnf exon IV* and *c-Fos* was performed using chromatin from $DAXX^{Flox/WT}$ and $DAXX^{Flox/Flox}$ cortical neurons infected with CRE lentivirus. Cells were left untreated or treated with 50mM KCl for 3 hours. ChIP with rabbit non-specific IgG was used as background control. Data are mean ± SEM (n = 3; n.s. non specific).

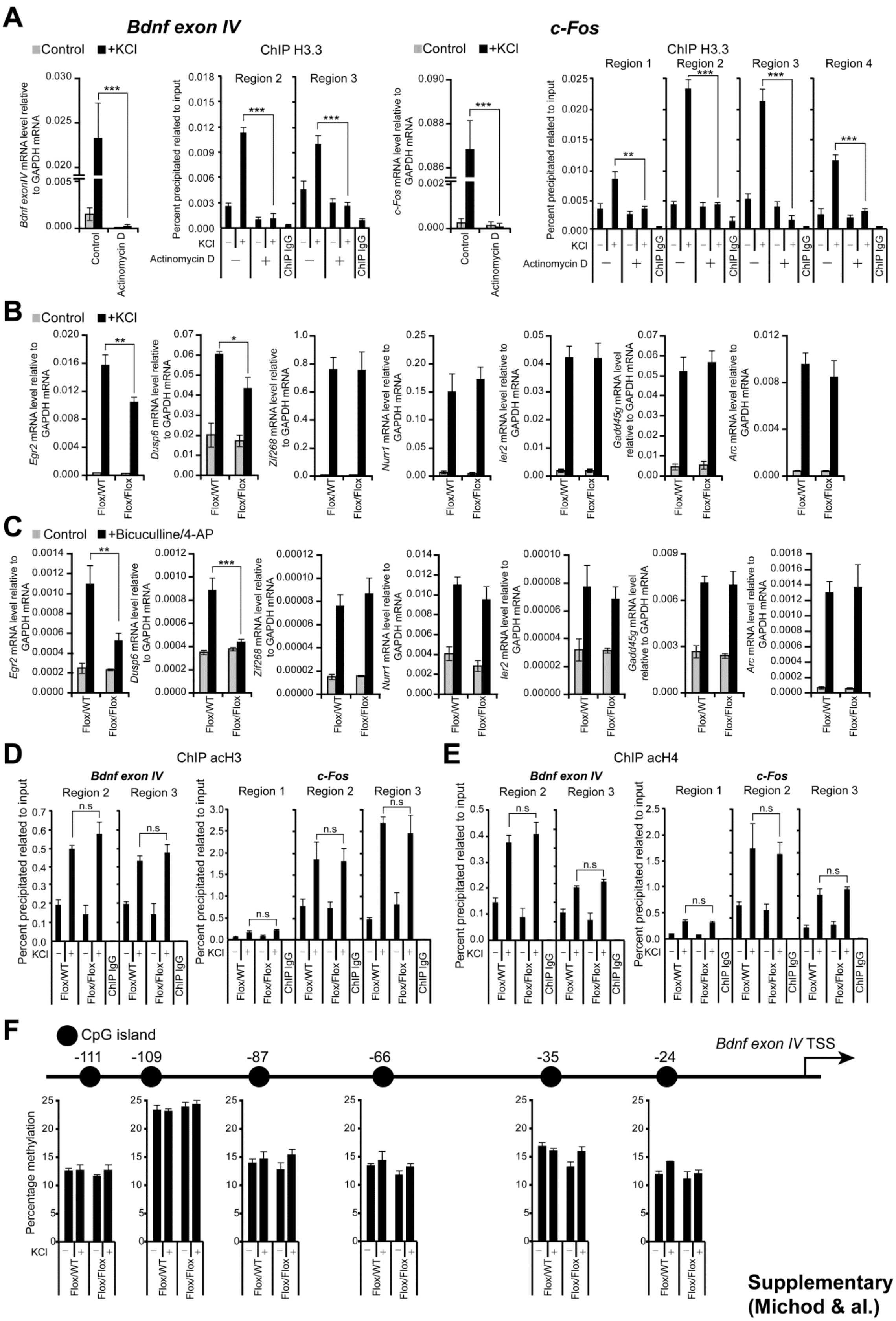

Supplementary Figure 4
(Michod & al.)

Figure S4 (related to Figure 4). DAXX regulates transcriptional induction of immediate early genes without affecting DNA methylation or histone acetylation

(A) qPCR analysis and ChIP analysis of H3.3 enrichment at the regulatory regions of *Bdnf exon IV* and all regions analyzed in Figure 2 for *c-Fos* were performed using cortical neurons DIV5. Cells were left untreated or treated with actinomycin D for 1 hour prior to KCl treatment. ChIP with rabbit non-specific IgG was used as background control. Data are mean ± SEM (n = 3; $^{**}P$<0.01, $^{***}P$<0.001; Two-Way ANOVA test with Bonferroni post-test). (B) $DAXX^{Flox/WT}$ and $DAXX^{Flox/Flox}$ cortical neurons were infected with lentivirus encoding CRE recombinase. qPCR for the expression of indicated genes was performed from RNA extracted at DIV5 from cells either untreated or membrane-depolarized with 50mM KCl for 3hours. Data are mean ± SEM (n = 3; $*P$<0.05, $**P$<0.01; Two-Way ANOVA test with Bonferroni post-test). (C) $DAXX^{Flox/WT}$ and $DAXX^{Flox/Flox}$ cortical neurons were infected as above. qPCR expression was performed from RNA extracted at DIV9 from cells either untreated or treated with 50 μM bicuculline and 2.5 mM 4-AP for 3hours. Data are mean ± SEM (n = 3; $**P$<0.01, $***P$<0.001; Two-Way ANOVA test with Bonferroni post-test). (D and E) ChIP analysis of histone 3 acetylation (D) and histone 4 acetylation (E) enrichment at the regulatory regions of *Bdnf exon IV* and *c-Fos* using chromatin from $DAXX^{Flox/WT}$ and $DAXX^{Flox/Flox}$ cortical neurons infected with CRE lentivirus. Cells were left untreated or treated with 50mM KCl for 3 hours. ChIP with rabbit non-specific IgG was used as background control. Data are mean ± SEM (n = 3; n.s. non specific. Two-Way ANOVA test with Bonferroni post-test). (F) Bisulfite sequencing analysis performed on 6 CpG sites near the transcription start site of *Bdnf exon IV*. DNA methylation was analyzed from untreated and depolarized cortical neurons DIV5 infected with CRE lentivirus. Data are mean ± SEM (n = 3).

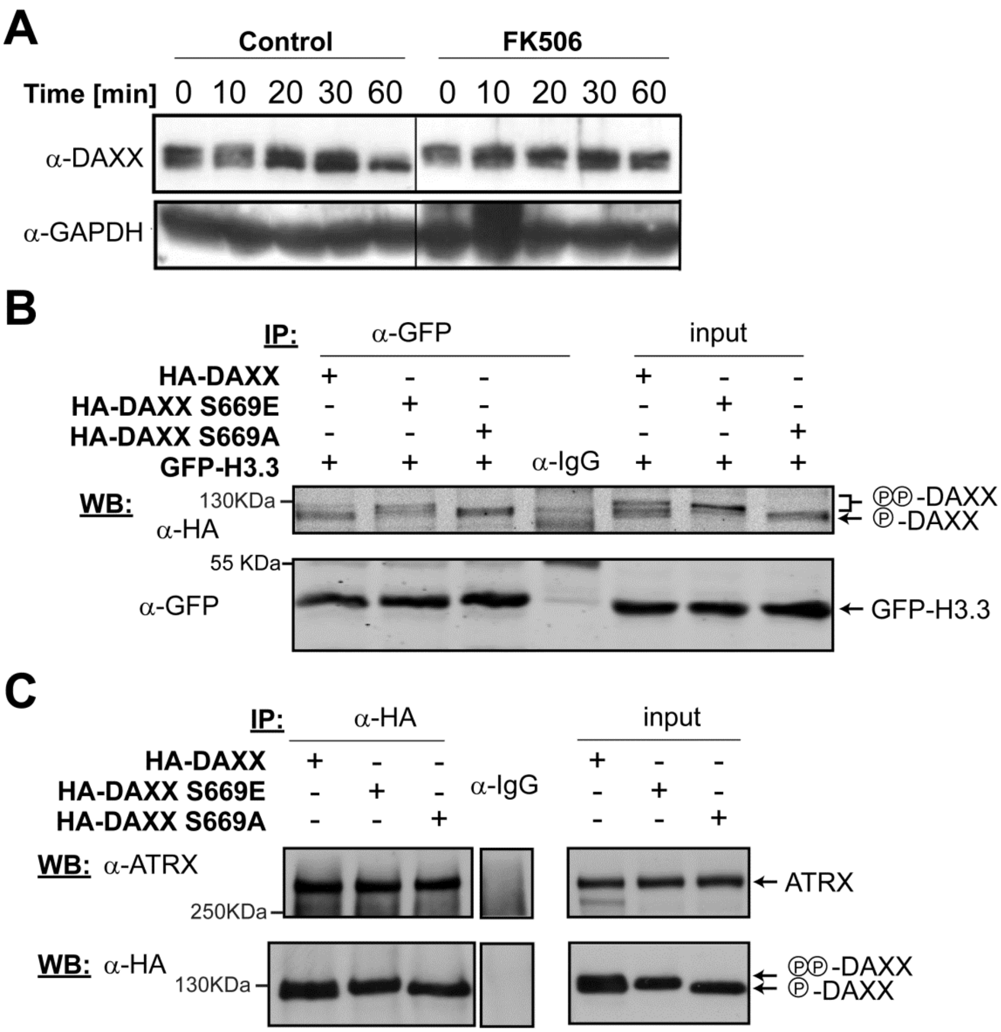

Supplementary Figure 5 (Michod & al.)

Figure S5 (related to Figure 5). Effect of calcium-dependent DAXX dephosphorylation on interaction with H3.3 and ATRX

(A) DIV11 cortical neurons were treated with 50 μM glutamate in the absence or presence of 100 nM FK506. Extracts were collected at the indicated time points and probed with α-DAXX and α-GAPDH antibodies. (B) Interaction between H3.3 and HA-DAXX (wild type), HA-DAXX S669E (phospho-mimetic), HA-DAXX S669A (phospho-mutant) in cortical neurons DIV4 cells. Extracts from cortical neurons cells infected with the indicated constructs were immunoprecipitated (IP) with anti-GFP or control antibody. The immunoprecipitates were analyzed by western blotting using the indicated antibodies. (C) Interaction between ATRX and HA-DAXX (wild type), HA-DAXX S669E (phospho-mimetic), HA-DAXX S669A (phospho-mutant) in cortical neurons DIV5 cells. Extracts from cortical neurons cells infected with the indicated constructs were immunoprecipitated (IP) with anti-HA or control antibody. The immunoprecipitates were analyzed by western blotting using the indicated antibodies.

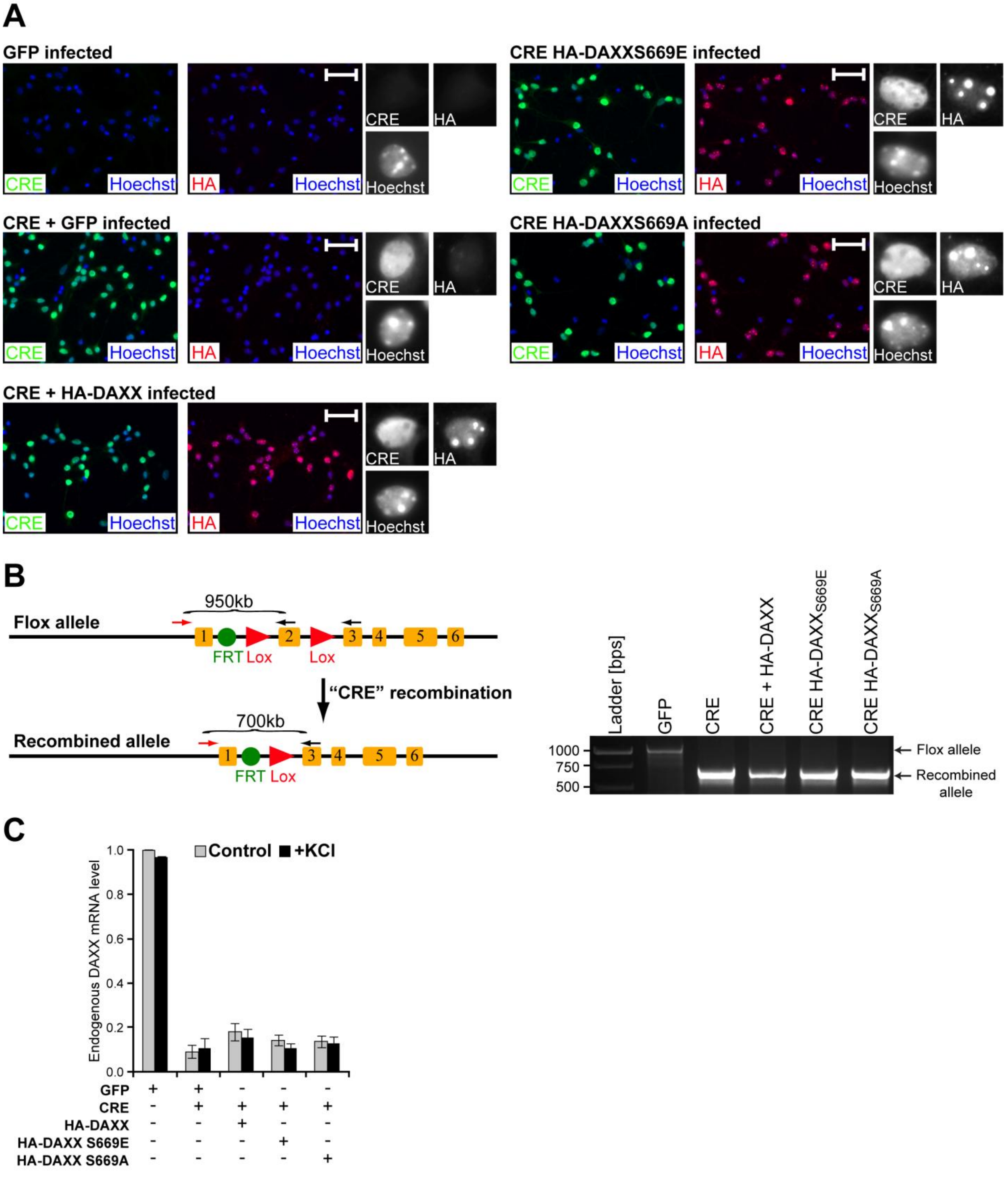

Supplementary Figure 6 (Michod & al.)

Figure S6 (related to Figure 6). Coinfection of cortical neurons DAXX$^{Flox/Flox}$ with CRE and HA-DAXX constructs results in expression of exogenous DAXX and abrogation of endogenous DAXX expression

(A) DIV5 *DAXX*$^{Flox/Flox}$ cortical neurons were infected with a GFP vector (MOI 1.75; control vector) or with combinations of CRE and HA-DAXX constructs [CRE/GFP (MOI 0.75/1.00); CRE/HA-DAXX (MOI 0.75/1.00)]. Immunofluorescence analysis was performed using α-HA and α-CRE antibodies. Scale bars represent 20μm. (B) Analysis of *DAXX* genomic locus in infected cells was performed using primers suitable for endogenous DAXX gene amplification (arrows), which allowed to discriminate endogenous DAXX alleles from integrated exogenous DAXX. (C) qPCR analysis of endogenous DAXX mRNA levels in *DAXX*$^{Flox/Flox}$ cortical neurons DIV5 infected as described in panel A, left untreated or treated with 50mM KCl for 3 hours.

Supplemental Experimental Procedures

Western Blot

Cells were washed in PBS, scraped in Laemmli Buffer and sonicated for 10 sec. Proteins were resolved on SDS-PAGE and transferred on nitrocellulose membrane. Membranes were blocked one hour in PBS 5% nonfat dry milk at room temperature and primary antibodies incubated O/N at 4ºC. The primary antibodies were detected by DyLight 680 or 800 conjugated secondary antibodies (Pierce) diluted 1:5,000 in PBS Tween 0.1% and 5% nonfat dry milk and subsequently visualized with the Odyssey® Infrared Imaging System (LI-COR). The antibodies specific for DAXX (Abcam #ab49287), ATRX (Santa Cruz Biotechnology #sc-15408), MeCP2 (Millipore # 07-013), CAF-1 p150 (Santa Cruz Biotechnology #sc-10772) and Lamin B (Santa Cruz Biotechnology #sc-20682) were diluted 1:1,000 and HA monoclonal antibody (Sigma-Aldrich #H9658) were diluted 1:2000 in PBS Tween 0.1% and 5% nonfat dry milk O/N at 4ºC.

Coimmunoprecipitation

Cells plated in a 10 cm petris dish were lysed in 500 µl of ice-chilled 100mM KCl, 5mM MgCl2, 20mM Tris-HCl pH 8, 0.1% Tween 20, 0.1% NP40, Complete® proteinase inhibitor (Roche), Complete® phosphatase inhibitor (Roche). Lysates were cleared by centrifugation at +4°C, and supernatants were incubated 2 hours with mouse antibodies specific for GFP (Roche #11814460001) or HA (Sigma #H9658) or mouse IgG. 40 µl of Protein G agarose beads were then added for an hour. Beads were washed 3 times with lysis buffer and finally resuspended in 50µl Laemmli buffer and heated at 95ºC for 5 min. Proteins were resolved on SDS-PAGE and transferred on nitrocellulose membrane. Membranes were blocked one hour in PBS 5% nonfat dry milk at room temperature and primary antibodies incubated O/N at 4ºC. The primary antibodies were detected by DyLight 680 or 800 conjugated secondary antibodies (Pierce) diluted 1:5,000 in PBS Tween 0.1% and 5% nonfat dry milk and subsequently visualized with the Odyssey® Infrared Imaging System (LI-COR). The antibodies specific for DAXX (Abcam #ab49287), GFP (Cell signaling #2555) and HA (Santa Cruz Biotechnology #sc-7292) were diluted 1:1,000

RNA Isolation, RT-PCR, and Quantitative Real-Time PCR Analysis

Total RNA was prepared using RNeasy kit (Qiagen). For reverse transcription, High Capacity RNA-to-cDNA Master Mix (ABI) was used and quantitative real-time PCR was performed in an Eppendorf Realplex Mastercycler using Maxima™ SYBR Green qPCR Master Mix (Fermentas). Relative abundance of the specific mRNAs was normalized to GAPDH mRNA. Primer sequences are in Supplemental table1.

In Vitro Phosphatase Assay

293T cells overexpressing the indicated constructs were lysed in 100mM KCl, 5mM $MgCl_2$, 20mM Tris-HCl pH 8.0, 0.1% Tween 20, 0.1% NP40, Complete® proteinase inhibitor (Roche). Lysates were cleared by centrifugation at +4°C, and the supernatant was incubated 2 hours with monoclonal HA antibody (Sigma-Aldrich # H9658). 50 µl of Protein G agarose beads were then added for an hour. Beads were washed 3 times with lysis buffer. Beads were then resuspended in calcineurin phosphatase buffer (20mM Tris pH8.0, 100mM NaCl, 1.5mM $CaCl_2$, 6mM $MgCl_2$, 0.5 mg/ml BSA, 1mM DTT, 100nM okadaic acid, 25 µg/ml calmodulin) and incubated with 500U calcineurin (Enzo Life Sciences) for 1 hour at 30°C. Reactions were stopped by adding Laemmli buffer and heated at 95ºC for 5 min.

Virus Preparation and Infection

Lentiviral supernatants were prepared as described previously (Salmon and Trono, 2006). Briefly, 293T cells plated in 15cm petri dish were transfected using calcium phosphate. Culture supernatants were collected at 48 h after transfection and lentiviral particles were concentrated using PEG (System Bioscience). Virus titrations were performed as described previously on 293T cells by qPCR (Salmon and Trono, 2006). Cortical neurons were infected with lentiviral particles 8 hours after plating and medium changed the day after.

Immunohistochemistry

Mouse brains were fixed in 4% formaldehyde PBS, dehydrated and embedded in paraffin. Five µm sections were cut, mounted onto glass slides, and antigen-retrieved in citric acid (pH 6.0) for 20 min using a pressure cooker. Sections were then incubated 30 min with 1% goat serum PBS and probed with primary antibody diluted in 1% goat serum PBS (DAXX (Abcam #ab49287),

ATRX (Santa Cruz Biotechnology #sc-15408), MeCP2 (Millipore #07-013)). Biotinylated secondary antibody was applied from the ABC-Elite kit (Vector Laboratories). DAB was used as chromagen (ImmPACT, Vector Laboratories).

Immunofluorescence

Cortical neurons were grown on glass coverslips coated with poly D-lysine (Sigma-Aldrich). Cells were fixed with 2% paraformaldehyde PBS for 30 min, permeabilized with PBS 0.3% Triton X-100 for 10 min, and incubated 30 min with PBS 1% goat serum. Coverslips were incubated O/N with the primary antibody diluted in PBS 1% goat serum (DAXX (Abcam #ab49287), ATRX (Santa Cruz Biotechnology #sc-15408), CRE (Novagen #69050), HA (Sigma-Aldrich # H9658)). Coverslips were washed in PBS and then incubated 1 h with 1/500 dilution of Alexa Fluor secondary antibodies (Invitrogen). The coverslips were washed in PBS and labeled with 1 μg/ml Hoechst 33258 (Sigma-Aldrich) before being mounted. For endogenous DAXX immunostaining, brief antigen retrieval was applied by high-power microwave heating for 20 seconds prior to permeabilization. Images were captured with a Leica confocal microscope or Leica inverted microscope.

Table S1. Primers Sequences

Primers for qPCR
Bdnf exon IV
sense 5’-CAGGAGTACATATCGGCCACCA
antisense 5’-GTAGGCCAAGTTGCCTTGTCCGT
c-Fos
sense 5’-CAGCTATCTCCTGAAGAGGAAG
antisense 5’-CTTCTCATCTTCAAGTTGAT
Npas4
sense 5’-GCTATACTCAGAAGGTCCAGAAGGC
antisense 5’-TCAGAGAATGAGGGTAGCACAGC
Zif268
sense 5’-TCTGAATAATGAGAAGGCGATGGT
antisense 5’-ACAAAGTGTTGCCACTGTTGGGTG
Nurr1
sense 5’-AGGTTCCAGGCAAACCCTGACTAT
antisense 5’-ATCTTCTCTGCCCACCCTCTGAT
Ier2
sense 5’-ACTGTCCCTTCCTTGGCTTGGAGA
antisense 5’-GCCGAAACGCGAATGGTAGTGAAA
Gadd45g
sense 5’-CGGGAAAGCACTGCACGAACTTCT
antisense 5’-ATTCAGGACTTTGGCGGACTCGTA
Egr2
sense 5’-TCTGGCCCTAGAGCAGTGAATGGA
antisense 5’-TTGCTTTGTCCTGCCCGACATTGC
Dusp6
sense 5’-ACCAGGCTGCTTTCTTTCTGTGTG
antisense 5’-AATGGAGCAAATCTCTCCCTCCGT
Arc
sense 5’-ATCCTGCAGATTGGTAAGTGCCGA
antisense 5’-GCACGTAGCCGTCCAAGTTGTTCT

Primers for ChIP-qPCR
Bdnf exon IV
**Region 1**
Sense 5'-TCCTTTGTTTTGATCACATGCTCCA
Antisense 5'-AAAAGCTCAAGCGAGAAAGGCCT
**Region 2**
Sense 5'-CACTAAGCCCCCTTCCTCTAGAGCA
Antisense 5'-ACTTATTGGCTGGATTAGAGGGGCA
**Region 3**
Sense 5'-AAAGTGGGTGGGAGTCCACGAG
Antisense 5'-GGCTTCTGTGTGCGTGAATTTGC
**Region 4**
Sense 5'-AAGAGTCTAGAACCTTGGGGACCGG
Antisense 5'-CCAAGTTACTGCTTCTAGCCGGGA
**Region 5**
Sense 5'-GGTGAGAAGAGTGATGACCATCC
Antisense 5'-GTAGGCCAAGTTGCCTTGTCCGT
c-Fos
**Region 1**
Sense 5'-TCATGTGCCAGCCCTTTCTAGC
Antisense 5'-CACACCGTCAGACAACAAGGGA
**Region 2**
Sense 5'-TTCACAGTAAAGAACGGTGGGAGC
Antisense 5'-TGCATTCCCTGTCACAGGCA
**Region 3**
Sense 5'-TCCATATTAGGACATCTGCGTCA
Antisense 5'-CGGCTCTATCCAGTCTTCTCAGT
**Region 4**
Sense 5'-ACCGACCTGCCTGCAAGATCC
Antisense 5'-TGCCTTCAGCTCCACGTTGC
Npas4
**Region 1**
Sense 5'-ACATGGATTAGGGTGGTCTGAAGC
Antisense 5'-TCCCCAAAAGCCAAGGAAAG
**Region 2**
Sense 5'-GGGGTAGGGAAGGGATCATCTTAG
Antisense 5'-TCCTCCTGCTTGCTATTTCG
Zif268
sense 5’-CCCGTGCTGTTCCAGACCCTTGAAAT
antisense 5’-AAGTTCTGCGCGCTGGGATCTCT
Nurr1
sense 5’-TATTTCAGGGAGATCTGACGGGCT
antisense 5’-GGTGGACAGTGTCGTAATTCAGCA
Ier2
sense 5’-CCTGCGGTTCCTTTGTCCTTA
antisense 5’-TCACTTTGGTTTCCGACATGC
Gadd45g
sense 5’-CGCGCATCGGACTCTGGGAATCTTT
antisense 5’-CATTGTGCGATCCACGAACAGCAA
Egr2
sense 5’-GCAGGAGAGAGTCAGTGACGGATA
antisense 5’-GCAACCAAGTTGTTGGCTTCT
Dusp6
sense 5’-TGTATCCATTGAGACGCTGGCTGT
antisense 5’-TCGCCGGATTATTCAAGACTGGGT
Arc
sense 5’-ACACACCCAGGGCTTCCATCC
antisense 5’-GACTAATGTGCTCTGCTGCGCG

Supplemental Reference

Salmon, P., and Trono, D. (2006). Production and titration of lentiviral vectors. Curr. Protoc. Neurosci. *Chapter 4*, Unit.
